# Aroyl-*S*,*N*-ketene acetal-triarylamine bichromophores – intramolecular energy transfer and dual emission upon induced aggregation and encapsulation

**DOI:** 10.1039/d5ra09361a

**Published:** 2026-02-20

**Authors:** Abdelouahad El Abbassi, Julius Krenzer, Eugene P. Petrov, Lukas Biesen, Vera Vasylyeva, Sarah Merzenich, Ute Resch-Genger, Thomas J. J. Müller

**Affiliations:** a Division Biophotonics, Bundesanstalt für Materialforschung und-prüfung (BAM) Richard-Willstätter-Straße 11 D-12489 Berlin Germany ute.resch@bam.de; b Institut für Organische Chemie und Makromolekulare Chemie, Heinrich-Heine-Universität Düsseldorf Universitätsstraße 1 D-40225 Düsseldorf Germany ThomasJJ.Mueller@hhu.de; c Abteilung für Crystal Engineering, Institut für Anorganische Chemie und Strukturchemie, Heinrich-Heine-Universität Düsseldorf Universitätsstrasse 1 40225 Düsseldorf Germany

## Abstract

Aroyl-*S*,*N*-ketene acetal-based bichromophores are synthesized by a catalytic aryl amination and their photophysics are studied in the solid state, in ethanol–water mixtures inducing aggregation, and after encapsulation in polystyrene nanoparticles. The dye substitution pattern controls aggregation-induced emission and intramolecular energy transfer efficiency, resulting in single-band or dual fluorescence and a strong increase in fluorescence quantum yield upon particle encapsulation.

## Abstract 2

Aroyl-*S*,*N*-ketene acetal bichromophores show enhanced emission upon aggregation and encapsulation. Partial energy transfer is controlled by substitution pattern and leads to aggregation induced dual emission (AIDE).

## Graphical abstract

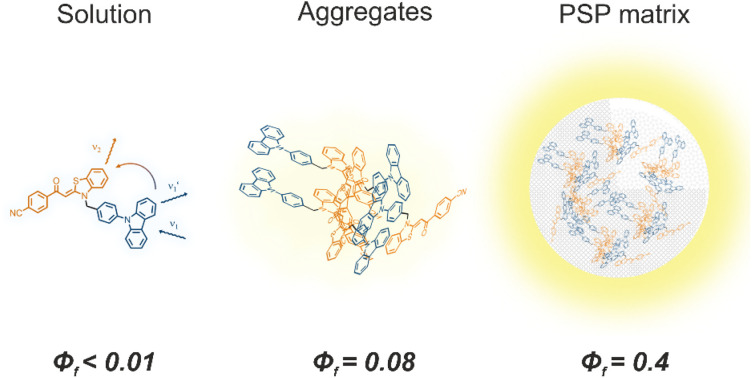

The discovery of aggregation-induced emission (AIE) challenged the long-held paradigm of aggregation-caused quenching (ACQ) and opened new opportunities for designing efficient luminescent materials,^1^ although numerous observations of the phenomenon had been made long before the term AIE was created.^2^ AIE-active luminogens (AIEgens) have now found application in sensing, bioimaging, and optoelectronics.^3–7^ Of particular interest are bichromophoric systems, where covalently linked donor–acceptor motifs reveal partial energy transfer (ET) resulting in dual emission—a property useful for color tuning, white-light generation, and sensing.^4–6^

While many AIE-active bichromophores have been reported up to date, aggregation-induced dual emission (AIDE) remains rare. Previous examples such as indolone-based merocyanines demonstrated that controlled aggregation can lead to white-light emission through partial ET.^7,8^ To this end, aroyl-*S*,*N*-ketene acetals have emerged as promising scaffolds owing to their tunable charge transfer character, facile synthetic modification, and relatively strong solid-state luminescence.^9,10^ However, until now, systematic studies of bichromophores derived from these acceptors and their potential for aggregation-controlled dual emission are still limited.

Here we present the design and photophysical investigation of four bichromophoric systems, referred to as dyes 3 (Scheme 1), that combine a blue emissive phenylcarbazole (PhCarb) or triphenylamine (TPA) donor with an orange-fluorescent aroyl-*S*,*N*-ketene acetal acceptor unit. Each of these luminophore moieties exhibits pronounced AIE. The observed aggregation-induced fluorescence enhancement can be attributed to either the restriction of intramolecular motions (RIM) that at least partly inhibits non-radiative depopulation pathways of the excited singlet state, or alternatively, to a restricted access to conical intersections (RACI) in the aggregated state.^11–13^ To assess potential ET and eventually AIDE, the photophysics of the bichromophores are studied in the solid state, in aggregation-inducing water/ethanol mixtures, and after encapsulation in polystyrene nanoparticles.

**Scheme 1:**
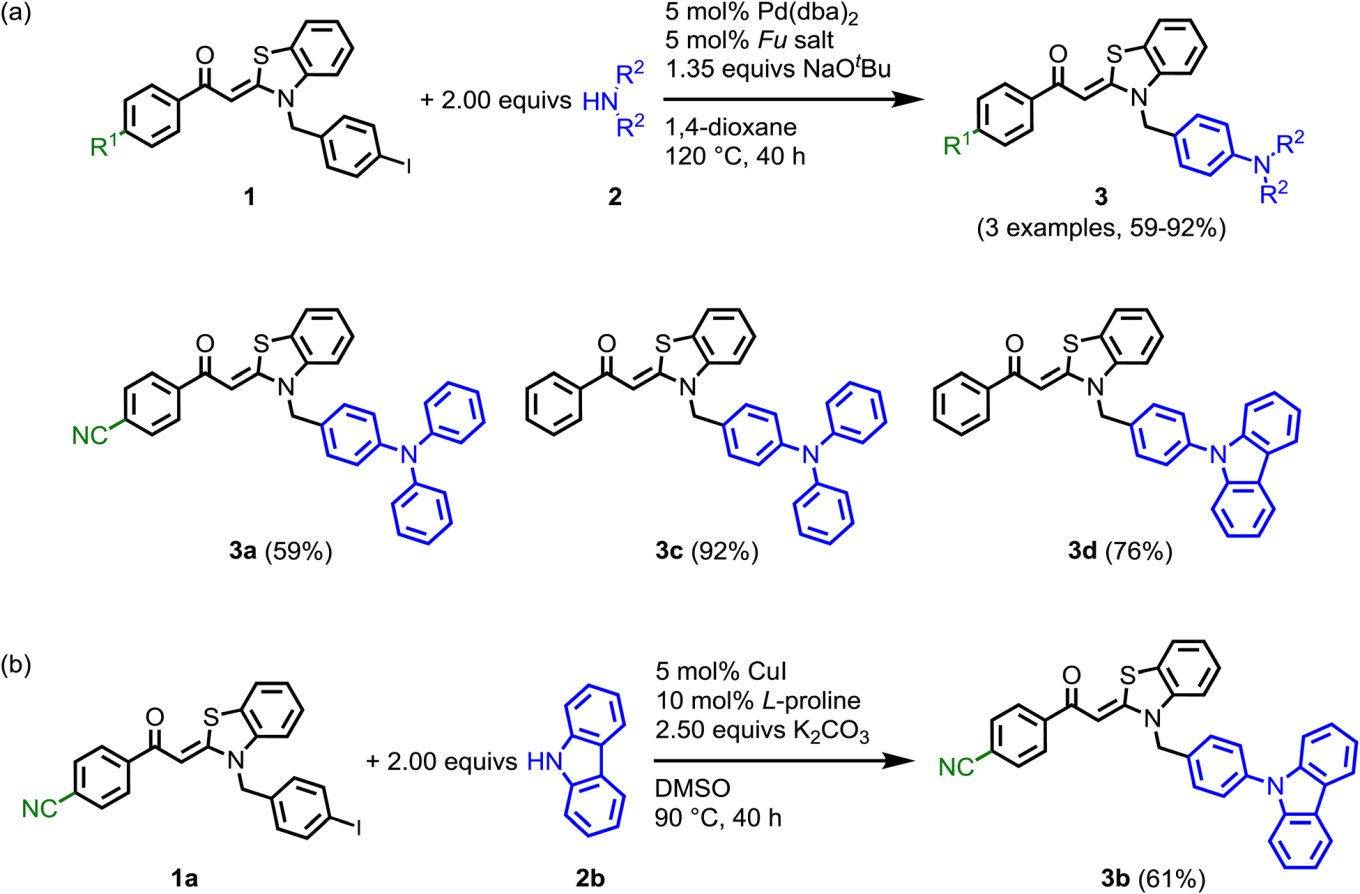
Synthesis of aroyl-*S*,*N*-ketene acetal based bichromophores 3 by Pd- (a) and Cu- (b) catalyzed aryl amination.

As shown in Scheme 1, two differently substituted aroyl-*S*,*N*-ketene acetals 1 (R^1^=H, CN) are used as starting materials for the synthesis of bichromophores 3.^14,15^ Similarly to Suzuki cross-coupling,^16^ iodo-functionalization at the *N*-benzyl substituents enables the substitution with different diarylamines 2*via* Buchwald–Hartwig amination using Pd(dba)_2_ and ^*t*^Bu_3_P·HBF_4_ as a catalyst system and NaO^*t*^Bu as a base (Scheme 1(a)). Since no product could be isolated in attempts to synthesize the cyano-substituted derivative 3b under these conditions, a copper-mediated Ullmann reaction was employed (Scheme 1(b)). The four bichromophores 3 were obtained after flash chromatography in moderate to excellent yields (Scheme 1). The molecular structures were confirmed by comprehensive NMR spectroscopy and mass spectrometry, and the chemical composition by combustion analysis and/or HRMS, respectively. The molecular structure of compound 3c was later corroborated by X-ray analysis (Fig. 1).^17^ The absence of strong intermolecular interactions (see SI) is consistent with the low melting point (102 °C) of the dye.

**Fig. 1:**
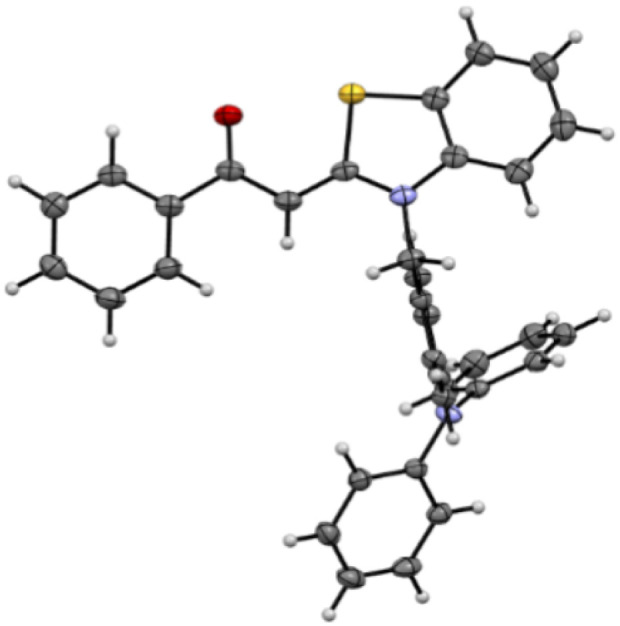
Molecular structure of dye 3c.

Absorption measurements of aroyl-*S*,*N*-ketene acetal bichromophore derivatives 3 in absolute ethanol revealed characteristic dual absorption profiles, with distinct maxima around 294 nm (*λ*_max,1_) and 402 nm (*λ*_max,2_) (see SI). The longer wavelength absorption band *λ*_max,2_ is attributed to electronic transitions within the aroyl-*S*,*N*-ketene acetal chromophore core,^14^ whereas the higher-energy absorption band *λ*_max,1_ corresponds to electronic transitions associated with the different blue-emitting electron-donating substituents incorporated into the bichromophoric systems. Dyes 3a, 3c, and 3d solely reveal an orange acceptor emission in pure ethanol independent of excitation wavelength, while 3b exhibits a blue emission from the PhCarb donor and a weak orange acceptor emission upon donor excitation (see Fig. 2, emission spectra measured at *f*_w_ = 0 for dyes 3a and 3b).

**Fig. 2:**
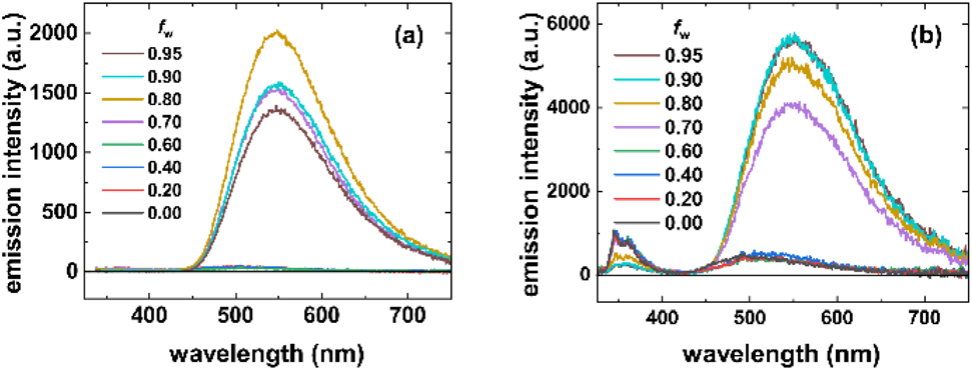
Emission spectra of 3a (a) and 3b (b) at different *f*_w_ showing complete and partial ET between the carbazole donor and the aroyl-*S*,*N*-ketene acetal acceptor chromophores, respectively, resulting in single band (a) and dual emission (b) upon water-induced dye aggregation (*λ*_exc_ = 290 nm).

All dyes 3 show a very low fluorescence efficiency in absolute ethanol with fluorescence quantum yields *Φ*_f_ < 0.01 independent of excitation at the donor or acceptor absorption bands. This indicates efficient non-radiative excited state depopulation of the dyes under these conditions. For all dyes, the formation of dye aggregates in ethanol/water mixtures is accompanied by distinct photophysical changes: (i) a bathochromic shift of the emission maximum of the orange fluorescence of the acceptor chromophore, (Fig. 2), and (ii) an enhancement of *Φ*_f_ as shown in Fig. 3a for dye 3b. We next carried out more detailed AIE studies with the water insoluble aroyl-*S*,*N*-ketene acetal bichromophoric dyes in ethanol/water solvent mixtures of increasing water volume fraction *f*_w_. All compounds exhibited a very weak fluorescence in solutions with *f*_w_ < 0.5, whereas at higher volume fractions of water (typically, *f*_w_ = 0.6⋯0.7), a sharp increase in the fluorescence efficiency was observed (see SI), which is typical for AIE and thus implies formation of dye aggregates in solution. Aggregated dyes 3a, 3c, and 3d always exhibited a single orange emission band originating from the acceptor chromophore upon excitation of the donor and acceptor moieties. This suggests very efficient ET in the aggregates. This behavior is depicted in Fig. 2a representatively for dye 3a.

**Fig. 3:**
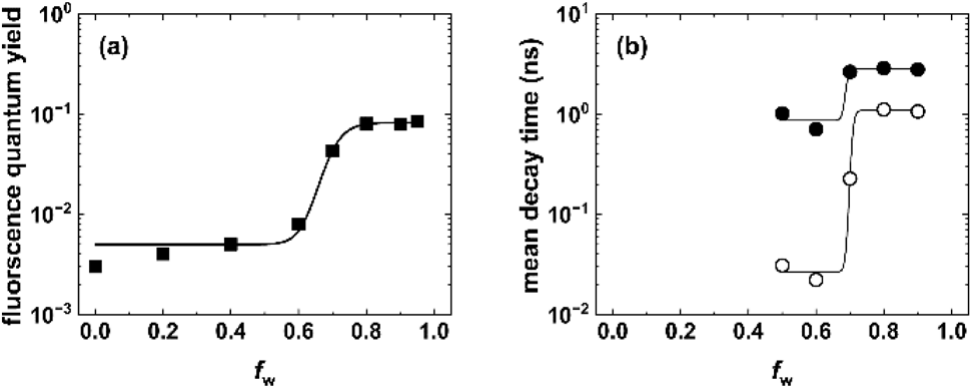
Fluorescence quantum yield (a) and mean fluorescence lifetime (b) as a function of the water content in ethanol/water mixtures. In (b), filled and open respectively. Lines are shown as a guide for the eye. ^*c*^(3b) = 10^−6^ M; *λ*_exc_ = *λ*^abs^_max_; *T* = 298 K.

In the aggregated state, reduced conformational freedom and shortened effective donor–acceptor distances can favor ET (energy transfer) processes. In addition to intramolecular donor–acceptor coupling, intermolecular interactions between neighboring bichromophores within the aggregates may further contribute to the high ET efficiency. As a result, the donor emission is efficiently quenched and the acceptor emission dominates the luminescence of aggregated dyes 3a, 3c, and 3d. However, dye 3b consisting of a PhCarb donor group and a nitrile electron withdrawing substituent on the acceptor moiety, behaves differently and exhibits exceptional AIE characteristics, *i.e.*, dual emission in the aggregated state for donor excitation at *λ*_exc_ = 290 nm with two distinct maxima at 348 (*λ*^em,1^_max_) and 500 nm (*λ*^em,2^_max_) as shown in Fig. 2b. This dual emission is ascribed to partial ET from the PhCarb donor to the aroyl-*S*,*N*-ketene acetal acceptor moiety whose absorption band less strongly overlaps with the donor fluorescence as is the case for dyes 3a, 3c, and 3d. Acceptor excitation of 3b at 400 nm leads to a low-intensity fluorescence with an emission maximum at 500 nm.

More detailed studies with dual emissive dye 3b revealed that at *f*_w_ < 0.5, excitation of the PhCarb chromophore at *λ*_exc_ = 290 nm results in a blue donor emission peaking at 348 nm almost independent of solvent polarity, and a very weak orange acceptor emission. However, at *f*_w_ > 0.5, excitation at 290 nm leads to an enhanced emission at 500 nm from the aroyl-*S*,*N*-ketene acetal chromophore. Excitation of the aroyl-*S*,*N*-ketene acetal acceptor at 400 nm yields the typical orange aroyl-*S*,*N*-ketene acetal fluorescence. The spectral position of this emission is affected by solvent polarity inducing a bathochromic shift with increasing *f*_w_ as shown for dyes 3a (Fig. 2a) and 3b (Fig. 2b). In contrast, the spectral position of the blue emission band of the PhCarb donor chromophore is not altered by microenvironment polarity (Fig. 2b). At a high-water content of *f*_w_ > 0.7, the emission profile of dye 3b undergoes a considerable change, and the acceptor band becomes dominant. Although the orange fluorescence of the acceptor is enhanced with increasing *f*_w_, nevertheless still dual emission of dye 3b is observed. However, the emission color observable by the eye develops a slightly more yellow touch.

A quantitative photophysical characterization corroborates these observations. Donor excitation at 290 nm results in *Φ*_f_ values of 0.02 (fluorescence integrated from 325 to 400 nm) for dye 3b at *f*_w_ = 0.9, while acceptor excitation at 400 nm yields *Φ*_f_ = 0.06 (fluorescence integrated from 425 to 750 nm) for the donor and acceptor moiety, respectively. *Φ*_f_ of the dual emission (integration from 325 to 750 nm) amounts to 0.08 for excitation at 290 nm. Additionally, performed time-resolved fluorescence measurements with dye 3b in water–ethanol mixtures (Fig. 3b), providing complementary fluorescence lifetime data, support our steady-state observations.

To reliably assess donor–acceptor spectral overlap, normalized emission spectra of isolated donor model compounds and absorption spectra of the corresponding acceptor chromophores were used. Compounds 3a and 3c, bearing a TPA donor, exhibit broader and slightly red-shifted donor emission bands relative to the PhCarb donors of 3b and 3d (Fig. 4). This broader TPA emission provides the basis for a larger spectral overlap with the acceptor absorption band for 3a and 3c. Furthermore, the cyano-substituted acceptor in 3a and 3b absorbs at longer wavelength. This enhances the spectral overlap in 3a but aligns less effectively with the narrower and more blue-shifted emission of the PhCarb donor in 3b. Still the emission bands of the series 3 are blue-shifted in comparison to previously reported similar bichromophores with biphenyl diarylamino/carbazole donors, which are mostly dominated by complete ET to the cyano-substituted acceptor.^10^

**Fig. 4:**
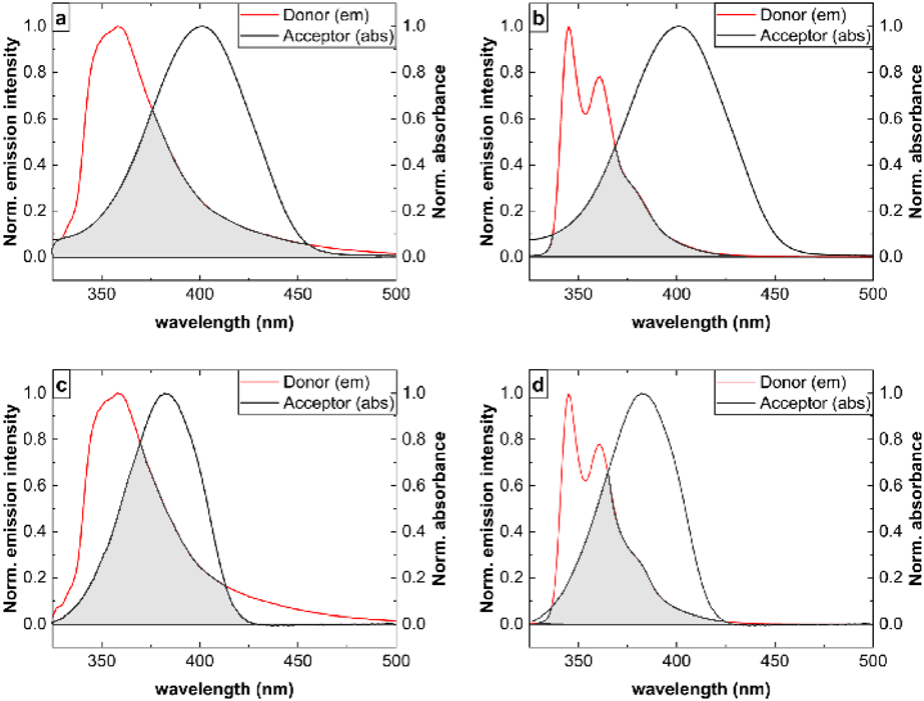
Normalized absorption spectra of the acceptor chromophores ((*Z*)-2-(3-benzylbenzo[*d*]thiazol-2(3*H*)-ylidene)-1-phenylethan-1-one and (*Z*)-4-(2-(3-benzylbenzo[*d*]thiazol-2(3*H*)-ylidene)acetyl)benzonitrile)^14^ and normalized fluorescence spectra of the corresponding donor model compounds (triphenylamine and *N*-phenyl carbazole) used to evaluate the spectral overlap for bichromophores 3a (a), 3b (b), 3c (c), and 3d (d) in ethanol. The spectra were recorded at a dye concentration of 10^−6^ M; *λ*_exc_ = 290 nm; *T* = 298 K. Donor fluorescence spectra were obtained by selective excitation of isolated donor chromophores (triphenylamine and *N*-phenyl carbazole), while the acceptor absorption spectra were recorded from the corresponding acceptor units ((*Z*)-2-(3-benzylbenzo[*d*]thiazol-2(3*H*)-ylidene)-1-phenylethan-1-one and (*Z*)-4-(2-(3-benzylbenzo[*d*]thiazol-2(3*H*)-ylidene)acetyl)benzonitrile). The spectral overlap between the absorption and emission bands of the donor and acceptor moieties of the dyes is highlighted in grey to emphasize its importance in controlling the efficiency of energy transfer (ET) in these molecules and thereby the observation of a single emission from the acceptor (complete ET) or dual emission from both moieties (partial ET). This determines the emission color of the dye aggregates observable by the eye.

The spectral position of the donor and acceptor emission and absorption bands together with the spectral width of the respective bands explain why 3b is the only bichromophoric dye of this series that shows dual emission also in the aggregated state, *i.e.*, revealing a blue donor emission and an orange acceptor emission upon aggregation (Fig. 4). The decreased spectral overlap between the donor emission and acceptor absorption band in 3b compared to 3a, 3c, and 3d reduces the ET efficiency too such an extent that the blue donor fluorescence can be still observed. This dual blue and orange fluorescence adds up to an overall white emission observable by the eye. In contrast, for 3a, 3c, and 3d, the broader donor emission bands, possibly in combination with a more favorable alignment and orientation of the donor–acceptor pairs including the respective transition dipole moments, result in a complete ET from the donor to the acceptor moieties. We can only speculate about the ET mechanisms involved. ET most likely occurs intramolecularly *via* a Förster type mechanism. However, in the aggregates also Dexter-type ET may be observed due to the shortened distances between donor and acceptor moieties of neighboring molecules.^18–22^

Next, we studied the effect of encapsulation into polystyrene particles (PSP) on the fluorescence behavior of our aroyl-*S*,*N*-ketene acetals. As previously demonstrated for different fluorophores including AIE dyes, this simple encapsulation concept, relying on premanufactured polymer particles with different surface functionalities, can be utilized to turn water-insoluble dyes lacking a reactive group into fluorescent reporters for life sciences applications by subsequent surface functionalization of the dye-loaded particles with recognition moieties or sensor molecules.^23^ In addition, the apolar polystyrene matrix protects fluorophores from the interaction with the environment, enhances their stability, and can improve their optical properties, especially their *Φ*_f_ by a combination of polarity and steric, *i.e.*, rigidization effects. Bichromophore 3b was selected as a model compound for these studies, based on its emission characteristics and AIE behavior. Dye encapsulation was achieved by a previously developed swelling protocol (see SI) using 200 nm diameter PSP. Fluorescence measurements of 3b-loaded PSP (Fig. 5) revealed dual band emission profiles similar to those observed in ethanol–water binary systems for excitation at 400 nm. As shown in Fig. 5, the lower polarity of PSP does not affect the spectral position and shape of the donor emission but induced a blue shift of the fluorescence of the acceptor moiety with its more pronounced CT character, originating from ET from the excited blue emissive donor to the acceptor. With its more pronounced CT character as well as a similar blue shift of its absorption band (see SI, Fig. S7), PSP, a partial ET from the blue emissive donor to the acceptor is observed (Fig. 5). The persistence of ET between the donor and acceptor moiety of 3b for different loading concentrations is ascribed to a slightly improved spectral overlap of the donor emission and acceptor absorption cause by a polarity induced hypsochromic shift of the acceptor absorption band in PSP (see SI, Fig. S7) preserving the intramolecular donor–acceptor coupling within the polymer matrix. These effects, together with the confinement of 3b in the rigid, apolar PSP environment, which restricts conformational freedom and fixes the distances and orientations between the donor and acceptor, could be beneficial for realizing a whitish appearance of the overall emission. In addition, a remarkable enhancement in *Φ*_f_ is observed upon PSP encapsulation, yielding values of 0.4 for the overall dual emission of 3b (integration of the fluorescence spectrum from 325 to 650 nm) in PSP. This value significantly exceeds the values obtained in pure ethanol, ethanol–water mixtures, and in the solid state. This considerable fluorescence enhancement is attributed to the rigid environment of the PSP system, which effectively suppresses non-radiative deactivation pathways through RIM.^24^

**Fig. 5:**
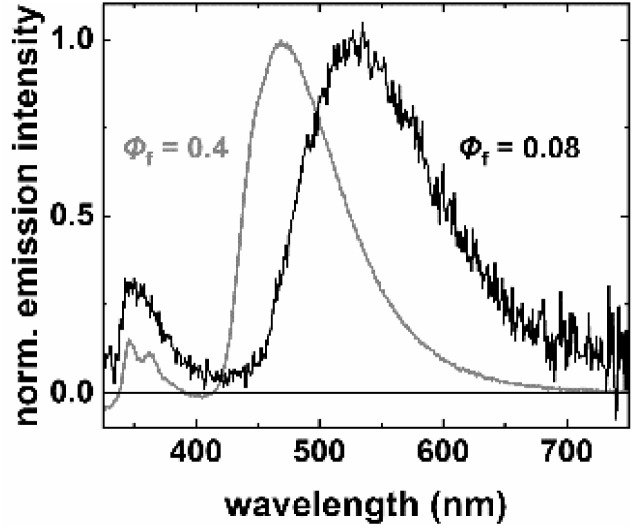
Normalized emission spectra of aggregates of dye 3b in ethanol/water solution (*f*_w_ = 0.9, black) and upon encapsulation in PSP (grey). Corresponding fluorescence quantum yields (*Φ*_f_) for both systems are indicated in the figure. *λ*_exc_ = 290 nm, *T* = 298 K.

In summary, we introduced aroyl-*S*,*N*-ketene acetal-triarylamine bichromophores which show aggregation-induced emission (AIE) in water–ethanol mixtures arising from donor–acceptor energy transfer (ET). All dyes displayed enhanced fluorescence in the aggregated state. The comparison of the absorption and emission properties of the four dyes and the fluorescence properties of their aggregates demonstrates the importance of the size of the spectral overlap between the donor fluorescence and acceptor absorption, which depends on the spectral position and spectral width of the respective bands and the environment sensitivity of these dye-specific features. For the observation of white light emission, these features need to be fine-tuned as shown for dye 3b, where the combination of the blue donor and the orange acceptor fluorescence leads to the visible observation of white fluorescence. A handle to fine-tune ET efficiency by the size of the spectral overlap of donor emission and acceptor absorption could present the combination of donor and acceptor moieties with a different CT character and hence a different response of their spectral absorption and emission properties to environment polarity. In addition, a remarkable increase in the quantum yield of the fluorescence of the representatively chosen dye 3b could be obtained by encapsulation into an apolar polymer matrix such as polystyrene particles (PSP). Despite the polarity-induced blue shift in absorption and fluorescence of the acceptor moiety of 3b, showing partial or frustrated ET in the aggregated state, still revealed dual emission also in PSP.

Overall, this underscores the potential of aroyl-*S*,*N*-ketene acetal based bichromophores as versatile building blocks for advanced photonic materials. Ongoing efforts are directed to expanding this dye library to further explore substitution effects, matrix encapsulation strategies, and conditions that could enable efficient white-light emission. These studies will deepen the understanding of AIE-driven dual emission and will lay the ground for practical applications in optoelectronics, sensing, and imaging.

## Author contributions

Synthetic studies, analytical characterization of the synthetic samples, photophysics (steady-state absorption and emission spectroscopy) by J. K., who compiled the obtained data. Methodologic and photophysical analytics (steady-state absorption and emission spectroscopy, time-resolved spectroscopy) by A. E. A., who compiled the obtained data. E. P. P. co-performed and evaluated time-resolved measurements and participated in data interpretation and manuscript revision. L. B. prepared first examples of bichromophores of the aroyl-*S*,*N*-ketene acetal series by Buchwald–Hartwig coupling and was involved in discussions. V. V. and S. M. performed the X-ray structure analysis, interpreted and compiled the data for Hirshfeld analysis. The conceptualization, project administration, funding acquisition, data interpretation and discussion, reviewing and editing by T. J. J. M. and U. R. G. The writing of the first draft was completed by J. K. and A. E. A., who contributed equally. All authors have read and agreed to the published version of the manuscript.

## Conflicts of interest

There are no conflicts to declare.

## Supplementary Material

RA-016-D5RA09361A-s001

RA-016-D5RA09361A-s002

## Data Availability

CCDC 2486717 contains the supplementary crystallographic data for this paper.^17^ The data supporting this article have been included as a part of the supplementary information (SI). Ref. 15 and 25–32 from the manuscript are cited in the SI as ref. 1–9. Supplementary information: synthetic, analytic, and spectroscopic details of compounds 3. See DOI: https://doi.org/10.1039/d5ra09361a.
