# Supplementary material for: Aroyl-S,N-ketene acetal-triarylamine bichromophores – intramolecular energy transfer and dual emission upon induced aggregation and encapsulation
Source: RSC Adv. 2026 Feb 20;16(11):10255–60. doi: 10.1039/d5ra09361a (PMC12922939; doi:10.1039/d5ra09361a)
Supplement: RA-016-D5RA09361A-s001 [file RA-016-D5RA09361A-s001.pdf]

Supplementary Information (SI) for RSC Advances.
This journal is © The Royal Society of Chemistry 2026

Electronic Supplementary Information for

Aroyl-*S*,*N*-ketene acetal-triarylamine bichromophores – intramolecular energy transfer and dual emission upon induced aggregation and encapsulation

Abdelouahad El Abbassi,[a] Julius Krenzer,[b] Eugene P. Petrov,[a] Lukas Biesen,[b] Vera Vasylyeva,[c] Sarah Merzenich,[c] Ute Resch-Genger*[a] and Thomas J. J. Müller*[b]

[a] Division Biophotonics, Bundesanstalt für Materialforschung und -prüfung (BAM), Richard-Willstätter-Straße 11, D-12489 Berlin, Germany.

[b] Institut für Organische Chemie und Makromolekulare Chemie, Heinrich-Heine-Universität Düsseldorf, Universitätsstraße 1, D-40225 Düsseldorf, Germany.

[c] Abteilung für Crystal Engineering, Institut für Anorganische Chemie und Strukturchemie, Heinrich-Heine-Universität Düsseldorf, Universitätsstrasse 1, 40225 Düsseldorf, Germany.

Table of Contents

1 **General considerations** 3

2 **Synthesis of aroyl-*S*,*N*-ketene acetal based bichromophores 3** 6

2.1 General procedure (GP) 6

2.2 Spectroscopic data 8

2.3 Synthesis of (*Z*)-4-(2-(3-(4-(9*H*-Carbazol-9-yl)benzyl)benzo[*d*]thiazol-2(3*H*)-ylidene)acetyl)benzonitrile (3b) 11

3 **NMR spectra** 12

3.1 NMR spectra of aroyl-*S*,*N*-ketene acetal based bichromophores 3 12

4 **Overview of photophysical properties of compounds 3** 16

5 **Absorption and emission spectra of compound** 17

6 **Spectral overlap table and calculation note** 23

7 **3b incorporation into polystyrene particles (PSP)** 24

8 **Crystal structure data** 25

9 **Literature** 30

1 General considerations

Reactions were carried out in dried and sintered Schlenk tubes or round bottom flasks under nitrogen atmosphere. Solvents were dried by a solvent purification system *MB-SPS-800* of the company *MBraun Inertgas-Systeme GmbH*.

The used chemicals, which have not been synthesized, were purchased at *Acros Organics BVBA, Alfa Aeser GmbH & Co KG, Fluorochem Ltd., J&K Scientific Ltd., Merck KGaA, Macherey-Nagel GmbH & Co. KG, Sigma-Aldrich Chemie GmbH* and *VWR* and have been used without further purification. The compounds for acceptor reference chromophores (aroyl-*S*,*N*-ketene acetals) were prepared according to literature and the UV/VIS spectra of (*Z*)-2-(3-benzylbenzo[*d*]thiazol-2(3*H*)-ylidene)-1-phenylethan-1-one (UV/Vis (acetone): $\lambda_{max}(\varepsilon)$ = 377 (39100)) and (*Z*)-4-(2-(3-benzylbenzo[*d*]thiazol-2(3*H*)-ylidene)acetyl)benzonitrile (UV/Vis (acetone): $\lambda_{max}(\varepsilon)$ = 397 (43600))[1] were used for creating the spectral overlap for assigning the energy transfer. The solvent ethanol (spectroscopic grade) was purchased from *Sigma-Aldrich* (Germany).

Purification: Was performed by flash column chromatography (silica gel M60 pore size 0.040-0.063 nm) of the company *Macherey-Nagel*. The crude product was adsorbed on Celite®545 of the company *Carl Roth GmbH*, placed on the suspended silica gel and purified with a positive pressure of 1 bar. Distilled solvent mixtures of *n*-hexane and acetone have been used as eluents.

Reaction progress: Was monitored by thin-layer chromatography (TLC) using silica gel 60 F254 aluminium plates (Macherey-Nagel GmbH & Co. KG).

Melting point determination: The melting points have been measured with *Melting Point B-540* of the company *Büchi*.

NMR: ^{1}H,^{13}C and DEPT 135-spectra have been measured at 298 K on an *Avance III - 300* and an *Avance III - 600* of the company *Bruker*. Chemical shifts in the ^{1}H and ^{13}C NMR are reported in ppm relative to deuterated solvents such as chloroform-d$_1$ (δ_H 7.26, δ_C 77.23), acetone-d$_6$ (δ_H 2.05, δ_C 29.84, δ_C 206.26) with CS_2 (δ_C 192.28) and DMSO-d$_6$ (δ_H 2.50, δ_C 39.51).[1] The multiplicity is abbreviated as followed: s = singlet; d = doublet; t = triplet; td = triplet of doublet; dd = doublet of doublet; dt = doublet of triplet, dq = doublet of quatet; pd = quintet of doublet m = multiplet. The assignment of primary carbon centers (CH), secondary carbon centers (CH_2), tertiary carbon centers (CH_3) and quaternary carbon centers (C_{quat}) were made by using DEPT-135 spectra.

Mass spectrometry: All mass spectrometry experiments have been performed by the department for mass spectrometry of the University of Düsseldorf (HHUCeMSA). EI mass spectra have been measured with Triple-Quadrupol-spectrometer *TSQ 7000* of the company *Finnigan MAT*. MALDI spectra have been measured with a *MALDI/TOF UltrafleXtreme* of the company *Bruker Daltronik*.

Infrared spectroscopy: IR spectra were recorded with neat compounds under attenuated total reflection (ATR) with *IRAffinity-1* of the company *Shimadzu* and the intensities were characterized as strong (s), middle (m) and weak (w).

CHN elemental analysis: The elementary analyses have been measured with *Perkin Elmer Series II Analyser 2400* or *Vario Micro Cube* of the company *Analysensysteme GmbH* at the microanalytical laboratory of the institute for Pharmaceutical and Medicinal Chemistry of the University Düsseldorf.

X-Ray Crystallography: For single-crystal X-ray diffraction (SCXRD), a suitable crystal was selected under a Leic M80 microscope and mounted on a cryo-loop using silicon oil. Diffraction data were collected with a Rigaku XtaLAB Synergy-S diffraction system equipped with a Hybrid Pixel Array 6000 detector and a Cu-Kα radiation (λ = 1.54184 Å) at 150(1) K. Cell refinement, data reduction, and absorption correction were executed with CrysAlisPro,[2] numeric absorption correction is performed using a multifaceted crystal model. OLEX2[3] was used to solve the crystal structures with SHELXT[4] and refined using SHELXL.[5] All the non-hydrogen atoms were refined with anisotropic displacement parameters. Hydrogen atoms involved in interactions were refined experimentally, while the remaining hydrogen atoms were positioned using the Uiso command. Crystallographic data for the structure have been deposited at the Cambridge Crystallographic Data Centre (CCDC Deposition Number 2486717). Important crystallographic data and refinement parameters are summarized in Table .

The supramolecular packing interactions have been analyzed by Hirshfeld surfaces using the program CrystalExplorer.[6]

Absorption: UV/Vis spectra of the dye solutions spectra were recorded on a double-beam spectrometer (Carry 5000) with a scan speed of 5 nm/s, an integration time of 0.2 s, and a slit width and step width of 1 nm.

Fluorescence: Fluorescence spectra were measured using a calibrated fluorometer FS5 (Edinburgh Instruments) equipped with a xenon lamp, using excitation and emission spectral bandwidths of 2 nm and 4 nm, the integration time of 0.5 s, a step width of 1 nm. The polarizers in the excitation and emission channel were set to 0° and 54.7° (magic angle conditions) to render the detected emission intensities independent of polarization effects. All spectra were corrected for the wavelength-dependent spectral responsivity of the fluorometer's detection channel.

The emission spectra of the solid compounds were recorded with a *Hitachi F-7000* spectrofluorometer using the emission correction curve provided by the instrument manufacturer.

Fluorescence quantum yields: The quantum yields (Φ), which represent the ratio of emitted photons to absorbed photons, were absolutely determined using an integrating sphere setup from Hamamatsu (Quantaurus-QY C11347-11). using special 10 mm × 10 mm long neck quatz cuvettes (Hamamatsu). The Quantaurus setup allows reliable measurement of Φ values ≥ 0.01.

Fluorescence decay kinetics: Fluorescence decay kinetics, providing the fluorescence lifetimes (τ), were recorded using a fluorometer FLS 920 (Edinburgh Instruments) equipped with an EPL405 excitation source and a fast multichannel plate photomultiplier (MCP-PMT) as the detector. All samples were excited at 405 nm, and the emission was detected at the emission maximum using a spectral bandwidth of the emission monochromator set to 15 nm. A 4096-channel setting and time ranges of 50 ns were utilized. This setup allows reliable measurement of τ values ≥ 0.2 ns. The measured fluorescence decay kinetics were evaluated using the reconvolution procedure of the F900 software, which considers the measured instrument response function influencing the fluorescence decays.

For all spectroscopic studies (absorption, emission, fluorescence decay kinetics, and quantum yield measurements), a saturated ethanolic stock solution of the dye (10^{-4} M, total volume 5 mL) was prepared. To investigate the AIE properties, aliquots of this stock solution were added to ethanol/water mixtures with increasing water content f_w ranging from 0,00 to 0,95. The mixtures were vortexed thoroughly and sonicated prior to measurement. All spectroscopic measurements were performed with air-saturated dye solutions at 25 °C using 10 × 10 mm quartz cuvettes (Hellma GmbH) filled with the respective solvent or dye solution.

2 Synthesis of aroyl-*S*,*N*-ketene acetal based bichromophores 3

The synthesis of aroyl-S,N-ketene acetals **1** has already been described in a previous publication.[7]

2.1 General procedure (GP)

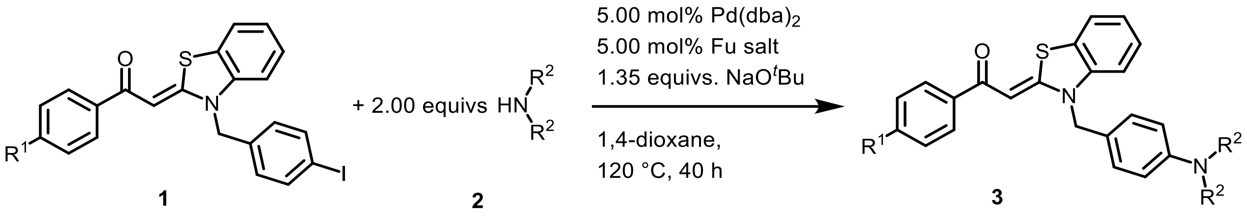

In a curved Schlenk tube aroyl-*S*,*N*-ketene acetal **1** (1.00 equiv), 5.00 mol% di(dibenzylideneacetone)palladium(0), 5.00 mol% Fu salt and sodium *tert*-butoxide (1.35 equiv) were dissolved in 1,4-dioxane (4 mL/mmol) (for experimental details, see Table S1). After addition of amine **2** (2.00 equiv), the mixture was stirred at 120 °C for 40 h. The catalyst was removed by filtration and after cooling the solution to room temperature, dichloromethane (30 mL), deionized water (30 mL), and saturated aqueous sodium sulfite solution (15 mL) were added to the mixture and extracted. The organic phase was separated and the aqueous phase was extracted with dichloromethane (3 x 20 mL). The combined organic phases were dried (anhydrous magnesium sulfate), filtered and the solvent was removed under reduced pressure. The product was adsorbed on Celite 545® and the solvent was removed under reduced pressure. This was followed by column chromatographic purification (n-hexane/acetone). For further purification of the compounds, the products were triturated with n-hexane, suspended in an ultrasonic bath, decanted and finally dried in a high vacuum.

Table S1: Experimental details for the synthesis of bichromophores **3**.

Entry	Aroyl-*S*,*N*-ketene acetal **1**	Amine **2**	$Pd(dba)_2$	Fu salt	NaO^tBu	Product **3**
**1**	148 mg (0.299 mmol) of **1a** (R^1 = CN)	102 mg (0.603 mmol) of **2a** (R^2 = Ph)	9.0 mg (0.016 mmol)	4.0 mg (0.015 mmol)	39 mg (0.41 mmol)	95 mg (59%) of **3a**
**2**[a]	141 mg (0.300 mmol) of **1b** (R^2 = H)	**2a** 61 mg (0.36 mmol) of **2a**	9 mg (0.016 mmol)	4.0 mg (0.015 mmol)	39 mg (0.41 mmol)	141 mg (92%) of **3c**

Table S1: Experimental details for the synthesis of bichromophores **3**.

Entry	Aroyl-*S*,*N*-ketene acetal **1**	Amine **2**	$Pd(dba)_2$	Fu salt	NaO^tBu	Product **3**
**3**	469 mg (0.999 mmol) of **1b** (R^2 = H)	334 mg (2.00 mmol) of carbazole (**2b**)	29 mg (0.050 mmol)	15 mg (0.052 mmol)	130 mg (1.35 mmol)	387 mg (76%) of **3d**

a:: *T* = 101 °C, *t* = 20 h.

2.2 Spectroscopic data

(*Z*)-4-(2-(3-(4-(Diphenylamino)benzyl)benzo[*d*]thiazol-2(3*H*)-ylidene)acetyl)benzonitrile (3a)

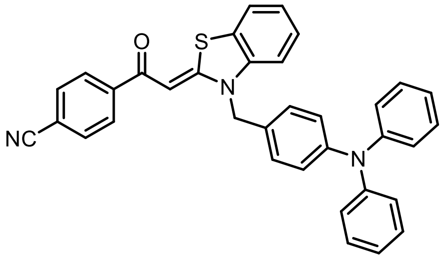

$C_{35}H_{25}N_3OS$

[535.67]

The synthesis was carried out according to **GP** and after flash chromatography on silica gel (*n*-hexane/acetone 5:1) compound **3a** (95 mg, 59%) was obtained as a yellow solid, Mp 187 °C, R_f (*n*-hexane/acetone 3:1): 0.36.

^{1}H NMR (300 MHz, DMSO-d$_6$): δ 5.65 (s, 2 H), 6.91 - 6.97 (m, 6 H), 6.98 - 7.04 (m, 2 H), 7.12 (s, 1 H), 7.20 - 7.31 (m, 7 H), 7.42 - 7.48 (m, 1 H), 7.56 (d, ^3J = 8.2, 1 H), 7.89 (dd, ^3J = 7.8 Hz, ^4J = 1.2 Hz, 1 H), 7.93 - 7.97 (m, 2 H), 8.15 - 8.19 (m, 2 H). ^{13}C NMR (75 MHz, DMSO-d$_6$): δ 48.0 (CH_2), 87.8 (CH), 111.6 (CH), 113.0 (C_{quat}), 122.8 (C_{quat}), 123.1 (CH), 123.4 (CH), 123.5 (CH), 123.8 (CH), 126.1 (C_{quat}), 127.1 (CH), 127.8 (CH), 128.1 (CH), 129.5 (CH), 129.5 (C_{quat}), 132.5 (CH), 139.5 (C_{quat}), 142.8 (C_{quat}), 146.7 (C_{quat}), 147.0 (C_{quat}), 162.5 (C_{quat}), 180.9 (C_{quat}). MALDI-TOF (*m/z*): 534.2 ([$C_{35}H_{25}N_3OS$ - H]$^+$), 258.2 ([$C_{19}H_{16}N$]$^+$). IR $\tilde{\nu}$ [cm^{-1}]: 2223 (w), 1973 (w), 1589 (m), 1557 (m), 1506 (m), 1474 (s), 1464 (s), 1420 (m), 1404 (m), 1391 (w), 1329 (m), 1315 (m), 602 (m), 633 (m), 696 (s), 747 (s), 795 (w), 812 (w), 820 (m), 851 (m), 881 (m), 918 (w), 930 (w), 970 (w), 1018 (m), 1111 (w), 1138 (w), 1157 (m), 1175 (m), 1194 (m), 1223 (m), 1279 (m), 1223 (m), 1194 (m), 1175 (m), 1157 (m), 1138 (w), 1111 (w), 1018 (m), 970 (w), 930 (w), 918 (w), 881 (m), 851 (m), 820 (m), 812 (w), 795 (w), 747 (s), 696 (s), 633 (m), 602 (m). UV/Vis (C_2H_5OH): λ_{max} (ε) = 297 (27400), 401 (28900). HRMS calcd. for [$C_{35}H_{25}N_3OS$ + H]$^+$: 536.1792; Found: 536.1791.

(*Z*)-2-(3-(4-(Diphenylamino)benzyl)benzo[*d*]thiazol-2(3*H*)-ylidene)-1-phenylethan-1-one (3c)

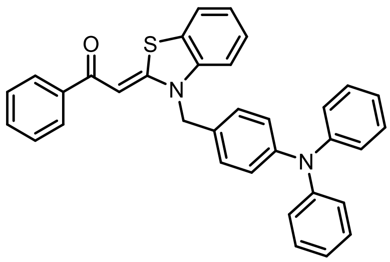

$C_{34}H_{26}N_2OS$

[510.18]

The synthesis was carried out according to **GP** and after flash chromatography on silica gel (*n*-hexane/acetone 3:1) compound **3c** (0.158 g, 92%) of product was obtained as a yellow solid, Mp 102 °C. R_f (*n*-hexane/acetone 3:1): 0.42.

^{1}H NMR (300 MHz, acetone-d$_6$): δ 5.57 (s, 2 H), 6.96 – 7.04 (m, 8 H), 7.10 – 7.14 (m, 1 H), 7.21 – 7.29 (m, 7 H), 7.38 – 7.50 (m, 5 H), 7.79 (dd, ^3J = 7.7 Hz, ^4J = 1.1 Hz, 1 H), 7.98 – 8.02 (m, 2 H). ^{13}C NMR (75 MHz, acetone-d$_6$): δ 49.3 (CH_2), 88.3 (CH), 111.6 (CH), 118.1 (CH), 121.0 (C_{quat}), 123.2 (CH), 123.8 (CH), 124.0 (CH), 124.5 (CH), 125.1 (CH), 127.6 (CH), 127.9 (C_{quat}), 128.0 (CH), 128.8 (CH), 129.1 (CH), 130.0 (CH), 130.2 (CH), 131.6 (CH), 140.7 (C_{quat}), 141.1 (C_{quat}), 148.4 (C_{quat}), 148.5 (C_{quat}), 162.5 (C_{quat}), 184.5 (C_{quat}). MALDI-TOF (*m/z*): 511.3 ($C_{34}H_{26}N_2OS+H^+$), 259.3 ($C_{19}H_{16}N+H^+$).IR $\tilde{\nu}$ [cm^{-1}]: 2972 (w), 2901 (w), 2824 (w), 1699 (w), 1591 (w), 1456 (s), 1439 (m), 1406 (m), 1381 (m), 1314 (m), 1229 (m), 1194 (w), 1175 (w), 1153 (w), 1074 (s), 1053 (s), 1028 (m), 1003 (m), 934 (w), 901 (m), 880 (m), 862 (m), 806 (w), 791 (w), 744 (m), 733 (m), 719 (m), 694 (m), 673 (m). UV/Vis (C_3H_6O): λ_{max} (ε) = 279 (48200), 382 (31900). Anal calcd. for $C_{34}H_{26}N_2OS$ [510.2]: C 79.97, H 5.13, N 5.49, S 6.28; Found: C 79.75, H 5.33, N 5.17, S 5.97.

(*Z*)-2-(3-(4-(9*H*-Carbazol-9-yl)benzyl)benzo[*d*]thiazol-2(3*H*)-ylidene)-1-phenylethan-1-one (3d)

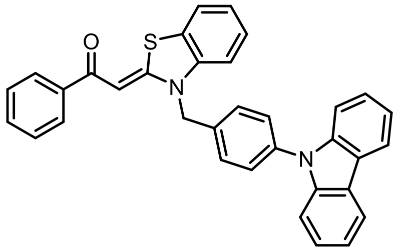

$C_{34}H_{24}N_2OS$

[508.16]

The synthesis was carried out according to GP and after flash chromatography on silica gel (*n*-hexane/acetone 4:1) compound **3d** (387 mg 76%) was obtained as a yellow solid, Mp: 228 °C. R_f (*n*-hexane/acetone 3:1): 0.43.

^{1}H NMR (300 MHz, acetone-d$_6$/CS_2 3:1): δ 5.74 (s, 2 H), 6.94 (s, 1 H), 7.21 – 7.33 (m, 3 H), 7.36 – 7.48 (m, 9 H), 7.60 – 7.67 (m, 4 H), 7.76 – 7.79 (m, 1 H), 7.96 – 8.00 (m, 2 H), 8.12 (dt, ^3J = 7.7 Hz, ^4J = 1.1 Hz, 2 H). ^{13}C NMR (75 MHz, acetone-d$_6$/CS_2 3:1): δ 49.4 (CH_2), 88.4 (CH), 110.4 (CH), 111.3 (CH), 121.0 (CH), 121.1 (CH), 123.3 (CH), 123.9 (CH), 124.2 (CH), 126.9 (CH), 127.5 (CH), 128.00 (CH), 128.03 (CH), 128.1 (C_{quat}), 129.0 (CH), 129.2 (CH), 131.5 (CH), 135.4 (C_{quat}), 137.9 (C_{quat}), 140.5 (C_{quat}), 140.8 (C_{quat}), 141.3 (C_{quat}), 162.4 (C_{quat}), 184.3 (C_{quat}). MALDI-TOF (*m/z*): 509.3 ($C_{34}H_{24}N_2OS+H^+$), 257.2 ($C_{19}H_{14}N+H^+$). IR $\tilde{\nu}$ [cm^{-1}]: 3057 (w), 2920 (w), 2851 (w), 2608 (w), 1701 (w), 1597 (m), 1570 (m), 1512 (m), 1477 (s), 1450 (s), 1437 (s), 1420 (m), 1379 (w), 1360 (m), 1335 (m), 1314 (m), 1294 (m), 1263 (m), 1225 (m), 1194 (m), 1169 (m), 1136 (w), 1117 (w), 1107 (w), 1094 (m), 1063 (m), 1043 (m), 1018 (m), 1001 (w), 966 (w), 924 (m), 912 (m), 878 (m), 851 (m), 818 (m), 806 (m), 791 (m), 746 (s), 719 (s), 692 (m), 673 (m), 648 (m), 635 (m), 621 (m), 806 (m), 791 (m), 746 (s), 719 (s), 692 (m), 673 (m), 648 (m), 635 (m), 621 (m). UV/Vis (C_3H_6O): λ_{max} (ε) = 281 (24200), 292 (23400), 339 (7200), 383 (27700). Anal calcd. for $C_{34}H_{24}N_2OS$ [508.2]: C 80.29, H 4.76, N 5.51, S 6.30; Found: C 80.09, H 4.92, N 5.26, S 6.30.

2.3 Synthesis of (*Z*)-4-(2-(3-(4-(9*H*-Carbazol-9-yl)benzyl)benzo[*d*]thiazol-2(3*H*)-ylidene)acetyl)benzonitrile (3b)

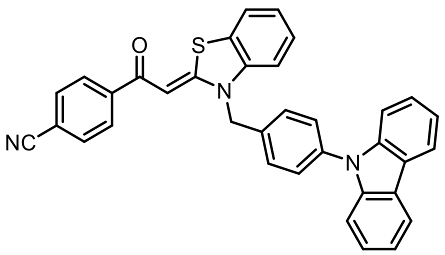

$C_{35}H_{23}N_3OS$

[533.65]

In a curved Schlenk tube aroyl-*S*,*N*-ketene acetal **1a** (148 mg, 0.299 mmol, 1.00 equiv), potassium carbonate (104 mg, 0.753 mmol, 2.51 equiv), copper iodide (3.0 mg, 0.016 mmol, 0.053 equiv), and *L*-proline (3.5 mg, 0.030 mmol, 0.10 equiv) were dissolved in DMSO (2 mL). After addition of carbazole (**2b**) (100 mg, 0.598 mmol, 2.00 equiv), the mixture was stirred at 90 °C for 40 h. The product was adsorbed on Celite 545® and the solvent was removed under reduced pressure. After flash chromatography (*n*-hexane/acetone 7:1 to 4:1) further purification of the product was achieved by trituration with *n*-hexane, suspension in an ultrasonic bath, decantation and finally drying in a high vacuum to give compound **3d** (97 mg, 61%) as a yellow solid, **Mp** 268 °C. R_f (*n*-hexane/acetone 3:1): 0.33.

^{1}H NMR (600 MHz, DMSO-d$_6$): δ 5.89 (s, 2 H), 7.21 - 7.28 (m, 3 H), 7.30 - 7.35 (m, 3 H), 7.35 - 7.40 (m, 2 H), 7.47 - 7.52 (m, 1 H), 7.54 - 7.58 (m, 2 H), 7.61 - 7.67 (m, 3 H), 7.93 - 7.99 (m, 3 H), 8.19 - 8.25 (m, 4 H). ^{13}C NMR (150 MHz, DMSO-d$_6$): δ 48.1 (CH$_2$), 87.8 (CH), 109.6 (CH), 111.6 (CH), 113.0 (C$_{quat}$), 118.6 (C$_{quat}$), 120.1 (CH), 120.5 (CH), 122.7 (C$_{quat}$), 122.9 (CH), 123.5 (CH), 126.2 (C$_{quat}$), 126.3 (CH), 127.0 (CH), 127.2 (CH), 127.8 (CH), 128.5 (CH), 132.5 (CH), 134.8 (C$_{quat}$), 136.2 (C$_{quat}$), 139.5 (C$_{quat}$), 139.9 (C$_{quat}$), 142.8 (C$_{quat}$), 162.7 (C$_{quat}$), 181.1 (C$_{quat}$). MALDI-TOF (*m/z*): 534.2 ([$C_{35}H_{23}N_3OS$ + H]$^+$), 256.1 ([$C_{19}H_{14}N$]$^+$). IR $\tilde{\nu}$ [cm^{-1}]: 3055 (w), 2988 (w), 2959 (w), 2901 (w), 2870 (w), 2224 (w), 1597 (m), 1560 (m), 1506 (m), 1477 (s), 1466 (s), 1449 (s), 1422 (m), 1408 (m), 1358 (m), 1323 (m), 1310 (m), 1300 (m), 1261 (w), 1225 (s), 1184 (m), 1171 (m), 1136 (w), 1111 (w), 1090 (m), 1067 (m), 1016 (m), 1003 (w), 970 (w), 912 (w), 876 (m), 854 (m), 833 (w), 814 (w), 799 (w), 741 (s), 721 (m), 710 (m), 700 (m), 677 (m), 642 (m). UV/Vis (C_3H_6O): λ_{max} (ε) = 291 (18700), 400 (18500). Anal calcd for $C_{35}H_{23}N_3OS$ [533.7]: C 78.78, H 4.34, N 7.87, S 6.01; Found: C 78.71, H 4.43, N 7.74, S 6.08.

3 NMR spectra

3.1 NMR spectra of aroyl-*S*,*N*-ketene acetal based bichromophores 3

^{1}H NMR spectrum (*Z*)-4-(2-(3-(4-(diphenylamino)benzyl)benzo[*d*]thiazol-2(3*H*)-ylidene)acetyl)benzonitrile (**3a**) (DMSO-d$_6$, 300 MHz, 298 K)

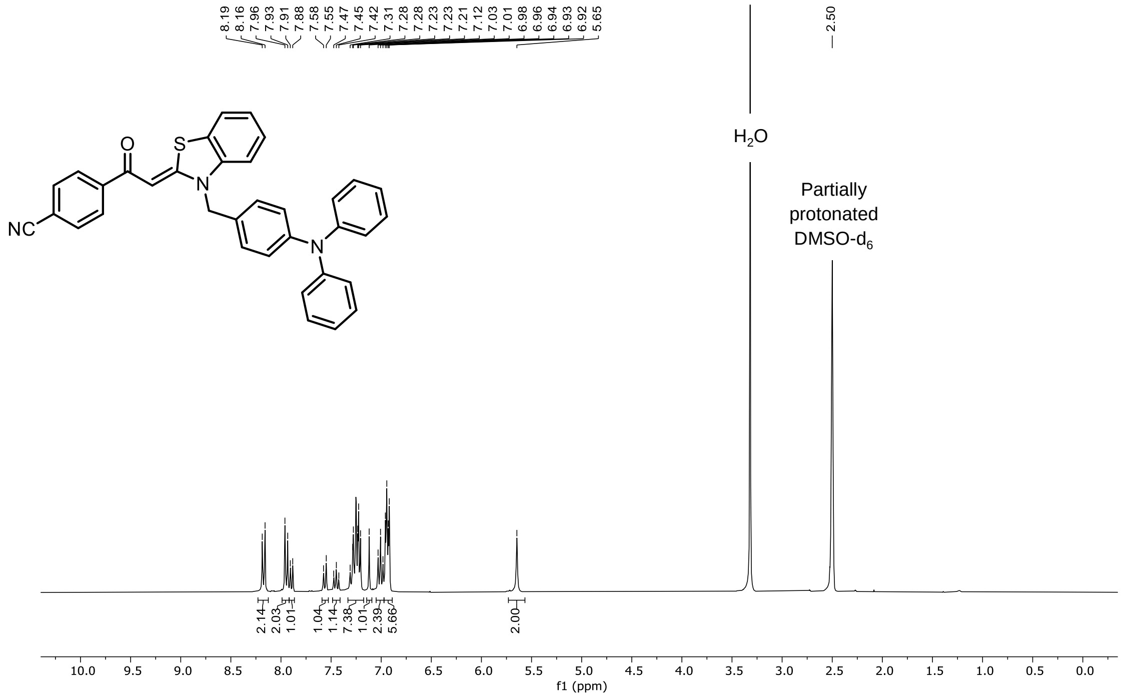

^{13}C NMR spectrum (*Z*)-4-(2-(3-(4-(diphenylamino)benzyl)benzo[*d*]thiazol-2(3*H*)-ylidene)acetyl)benzonitrile (**3a**) (DMSO-d$_6$, 75 MHz, 298 K)

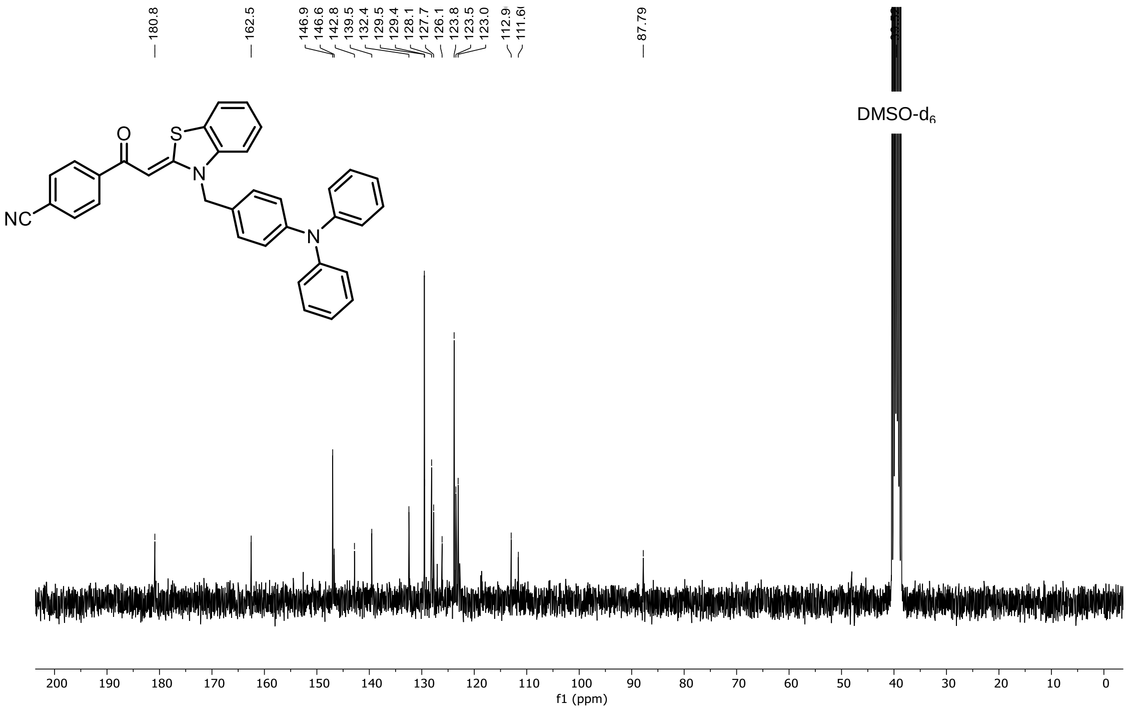

^{1}H NMR spectrum (*Z*)-4-(2-(3-(4-(9*H*-carbazol-9-yl)benzyl)benzo[*d*]thiazol-2(3*H*)-ylidene)acetyl)benzonitrile (**3b**) (DMSO-d_6, 600 MHz, 298 K)

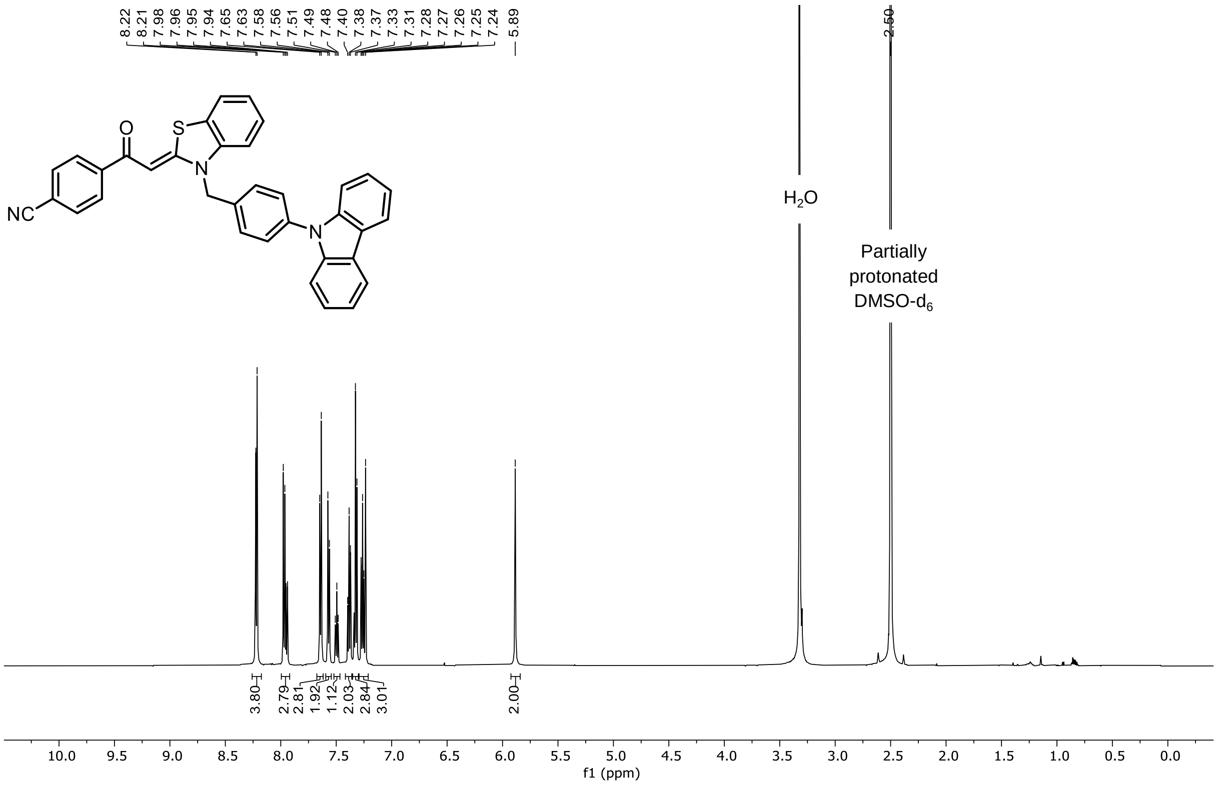

^{13}C NMR spectrum (*Z*)-4-(2-(3-(4-(9*H*-carbazol-9-yl)benzyl)benzo[*d*]thiazol-2(3*H*)-ylidene)acetyl)benzonitrile (**3b**) (DMSO-d_6, 150 MHz, 298 K)

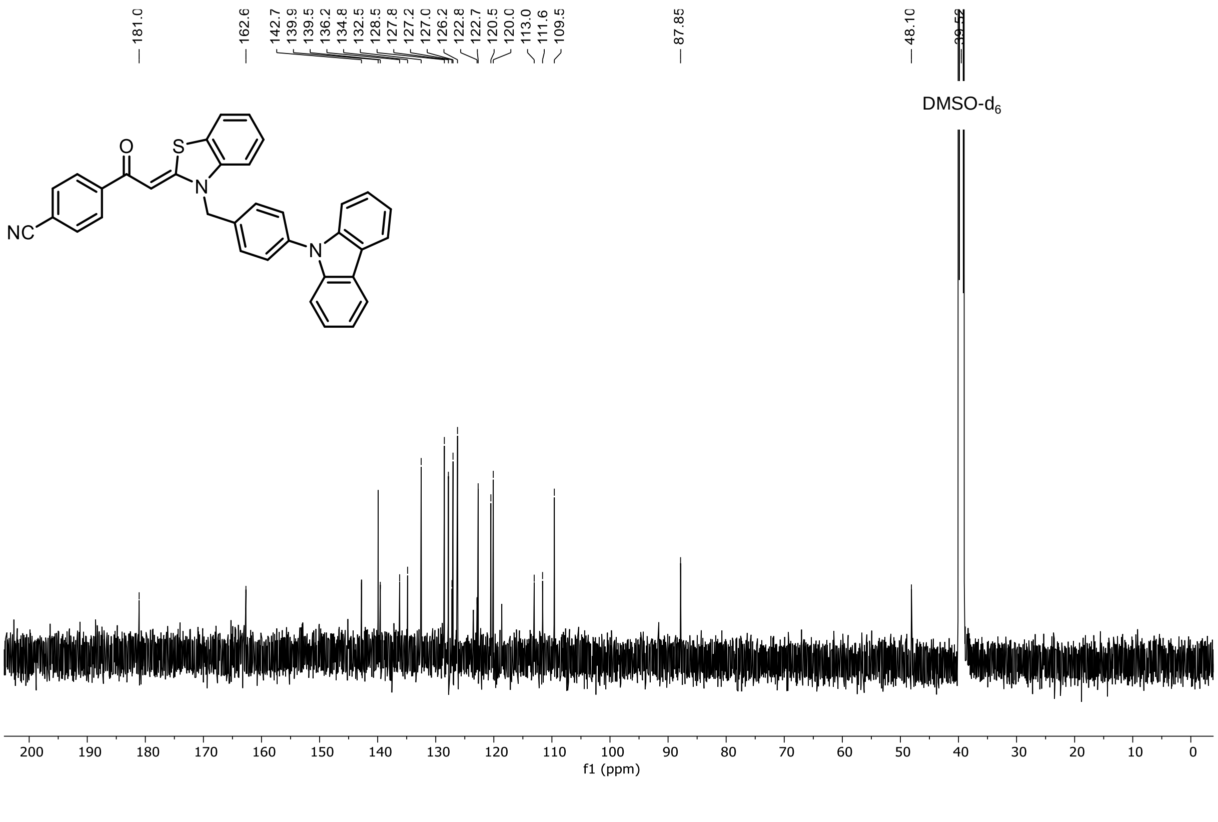

^{1}H-NMR spectrum (*Z*)-2-(3-(4-(diphenylamino)benzyl)benzo[*d*]thiazol-2(3*H*)-ylidene)-1-phenylethan-1-one (**3c**) (acetone-d$_6$, 300 MHz, 298 K)

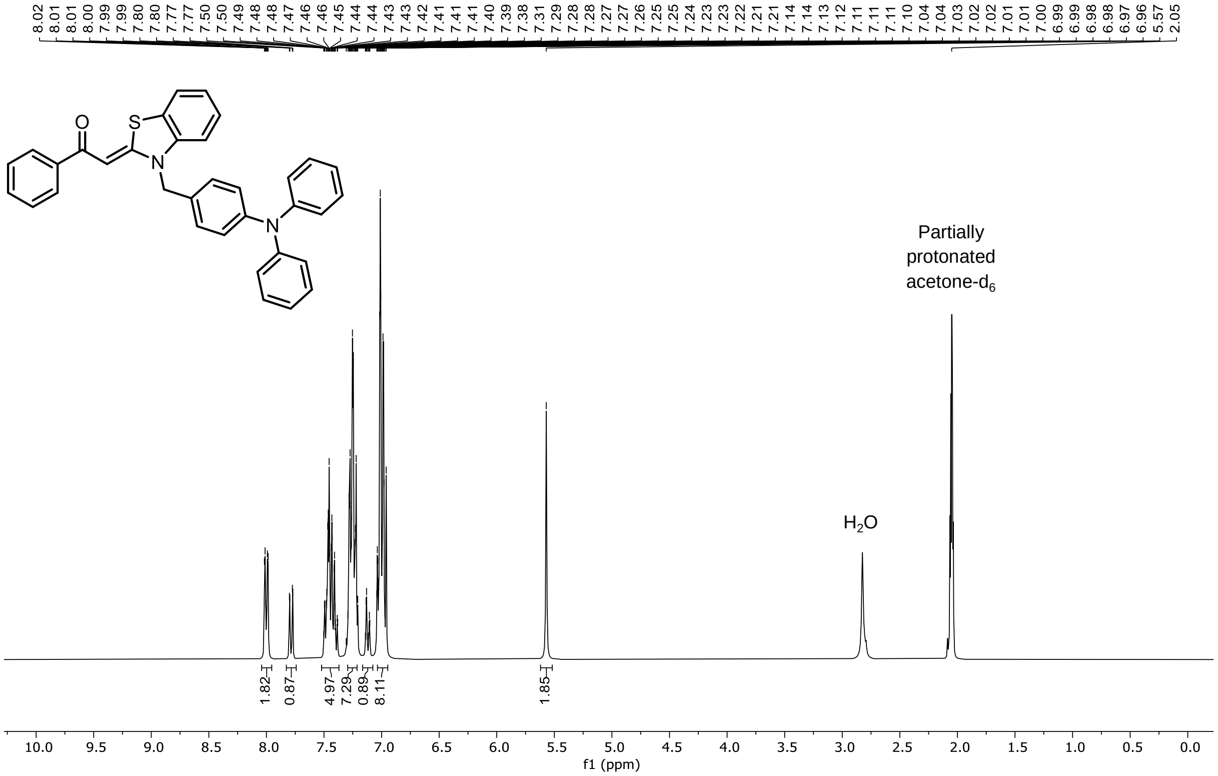

^{13}C NMR spectrum (*Z*)-2-(3-(4-(diphenylamino)benzyl)benzo[*d*]thiazol-2(3*H*)-ylidene)-1-phenylethan-1-one (**3c**) (acetone-d$_6$, 75 MHz, 298 K)

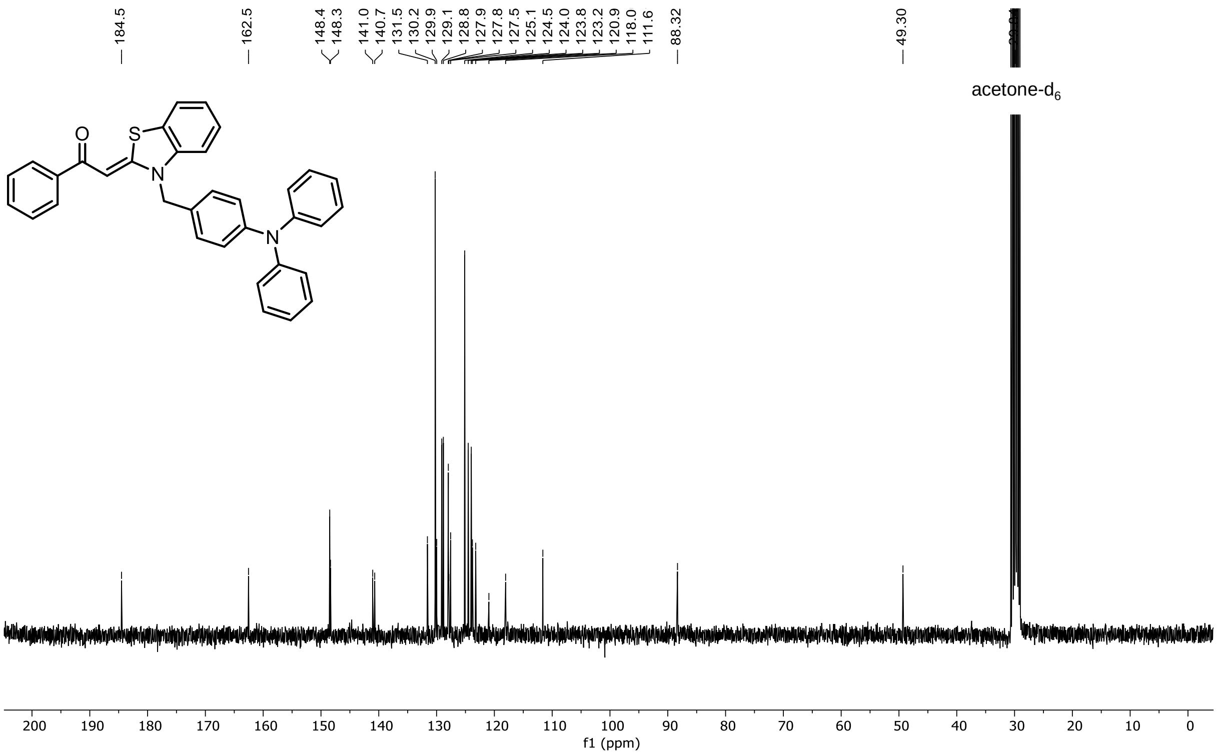

^{1}H NMR spectrum (*Z*)-2-(3-(4-(9*H*-carbazol-9-yl)benzyl)benzo[*d*]thiazol-2(3*H*)-ylidene)-1-phenylethan-1-one (**3d**) (acetone-d_6/CS_2 3:1, 300 MHz, 298 K)

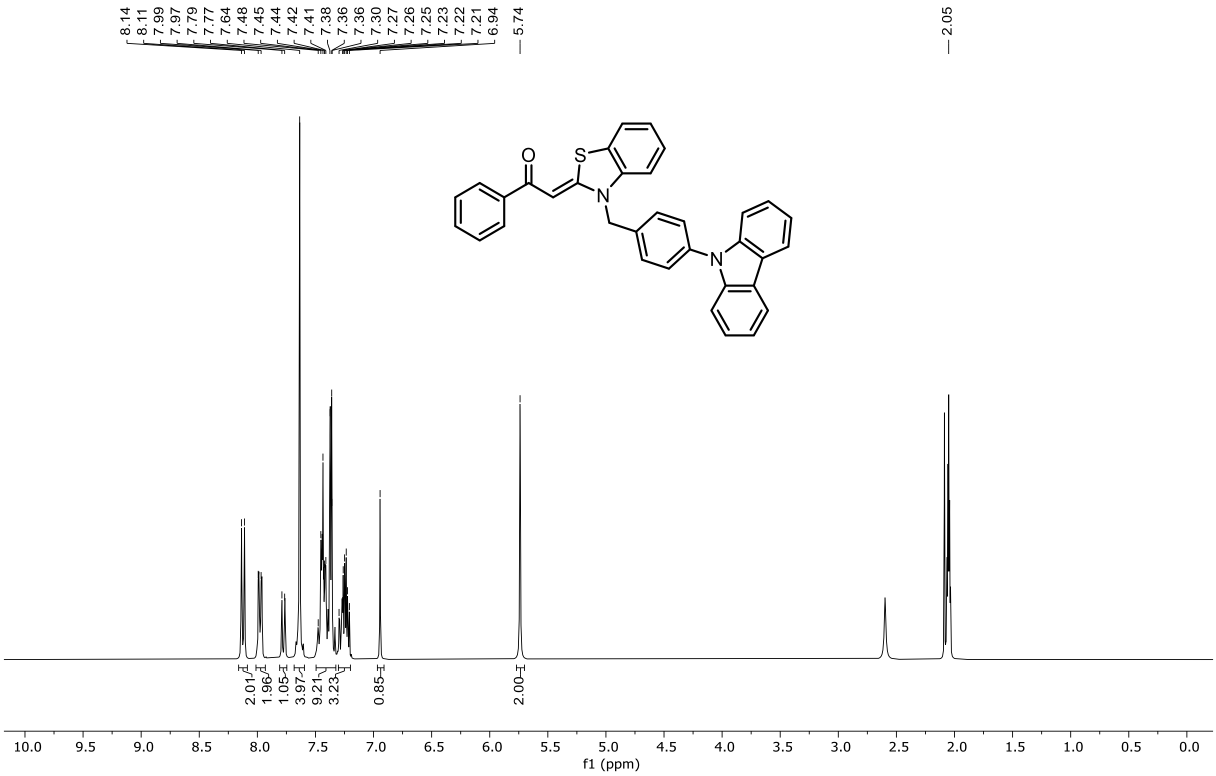

^{13}C NMR spectrum (*Z*)-2-(3-(4-(9*H*-carbazol-9-yl)benzyl)benzo[*d*]thiazol-2(3*H*)-ylidene)-1-phenylethan-1-one (**3d**) (acetone-d_6/CS_2 3:1, 75 MHz, 298 K)

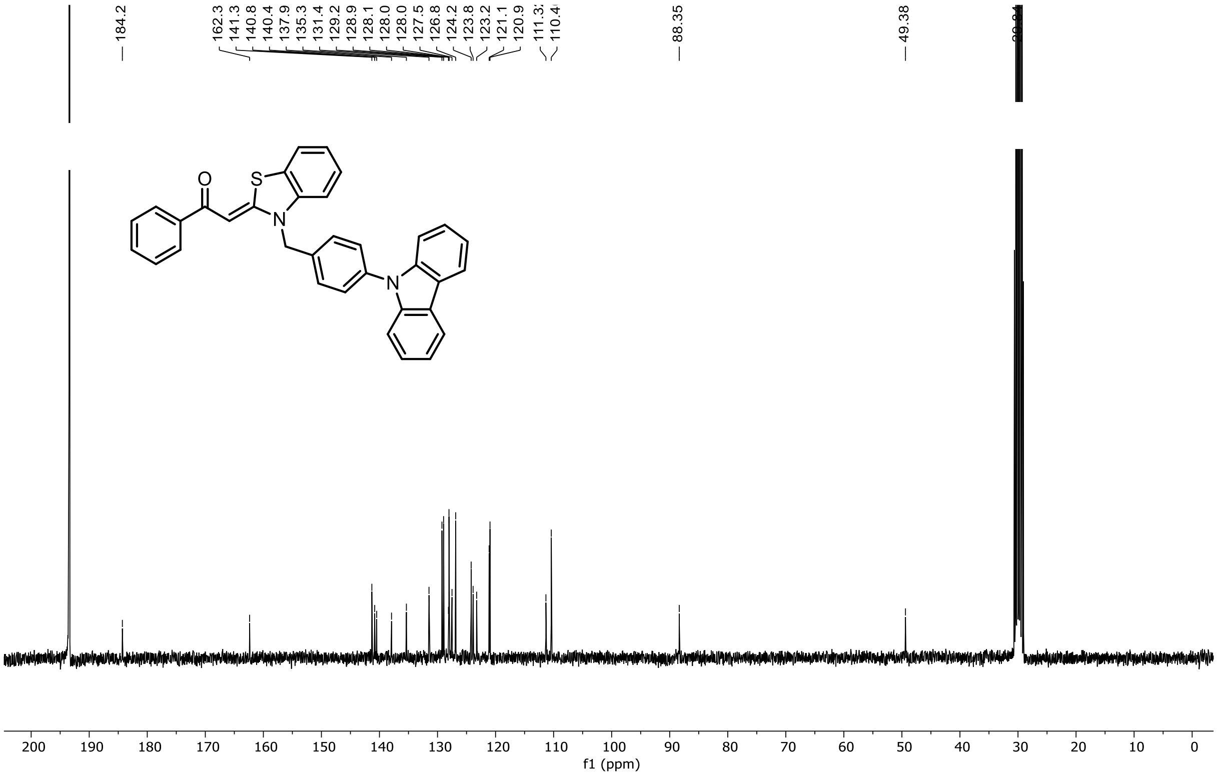

4 Overview of photophysical properties of compounds 3

Table S2: Photophysical properties of investigated aminated aroyl-*S*,*N*-ketene acetals **3** (absorption: measured in ethanol, T = 298 K, 10^{-5} M, emission: measured in ethanol ($f_w = 0$), in ethanol/water with a water content $f_w = 0.8$ (Aggregates), and is solid state (powder), (T = 298 K, 10^{-6} M, $\lambda_{\mathrm{exc}} = \lambda_{\mathrm{max}}^{abs}$).

Compound	$\lambda_{\mathrm{max}}^{abs}$ / nm (ε / L·mol^{-1}·cm^{-1})	$\lambda_{\mathrm{max}}^{\mathrm{em}}$ / nm			Φ_f		
		$f_w = 0$	$f_w = 0.8$	Solid State	$f_w = 0$	$f_w = 0.8$	Solid State
**3a**	298 (27400)<br>401 (28900)	358<br>496	550	530	<0.01	0.05	0.02
**3b**	291 (18700)<br>401 (18500)	345<br>500	348<br>550	512	<0.01	0.08	0.03
**3c**	298 (48200)<br>382 (31900)	359<br>450	490	504	<0.01	0.07	0.12
**3d**	291 (76200)<br>382 (27700)	345<br>450	490	480	<0.01	0.06	0.13

5 Absorption and emission spectra of compound

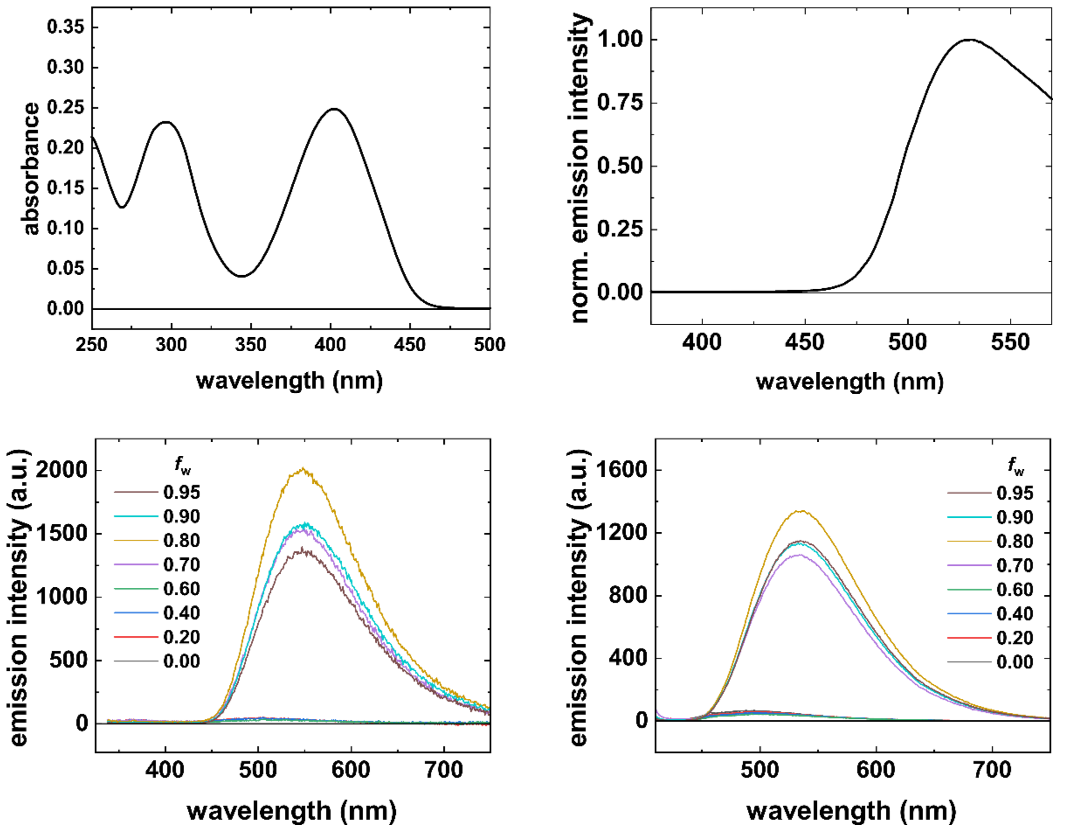

Figure S1: Absorption spectra of **3a** in ethanol (top, left), solid-state emission spectrum (top, right), AIE-related emission spectra of compound **3a** (2nd row) at different excitation wavelengths. (λ_{exc} = 298 nm (bottom left) ; λ_{exc} = 398 nm (bottom right))

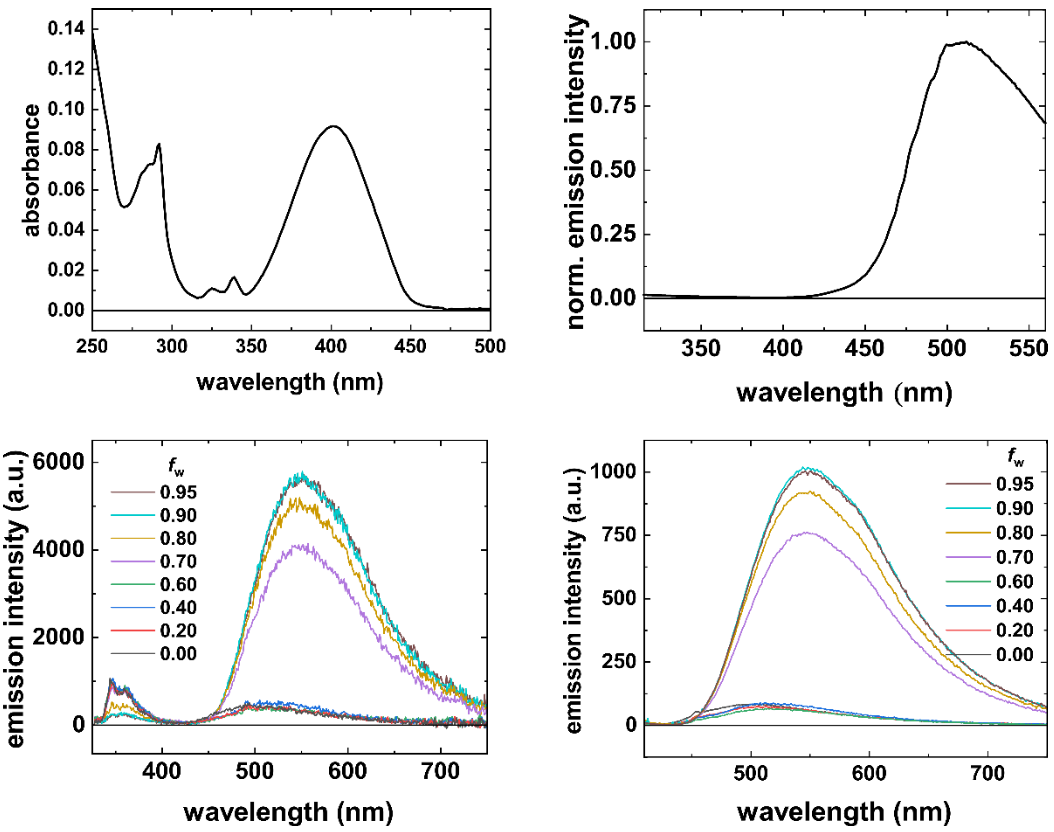

Figure S2: Absorption spectra of **3b** in ethanol (top, left), solid-state emission spectrum (top, right), AIE-related emission spectra of compound **3b** (2nd row) at different excitation wavelengths. (λ_{exc} = 290 nm (bottom left) ; λ_{exc} = 400 nm (bottom right))

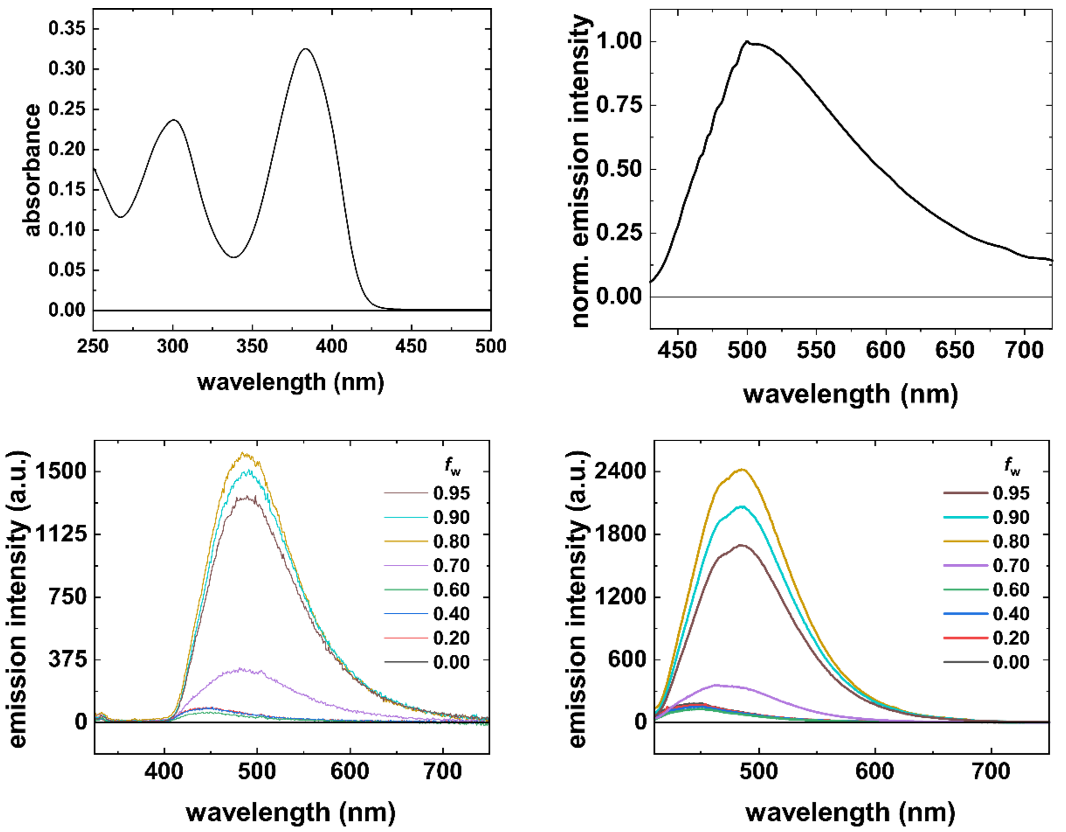

Figure S3: Absorption spectra of **3c** in ethanol (top, left), solid-state emission spectrum (top, right), AIE-related emission spectra of compound **3c** (2nd row) at different excitation wavelengths. (λ_{exc} = 298 nm (bottom left) ; λ_{exc} = 385 nm (bottom right))

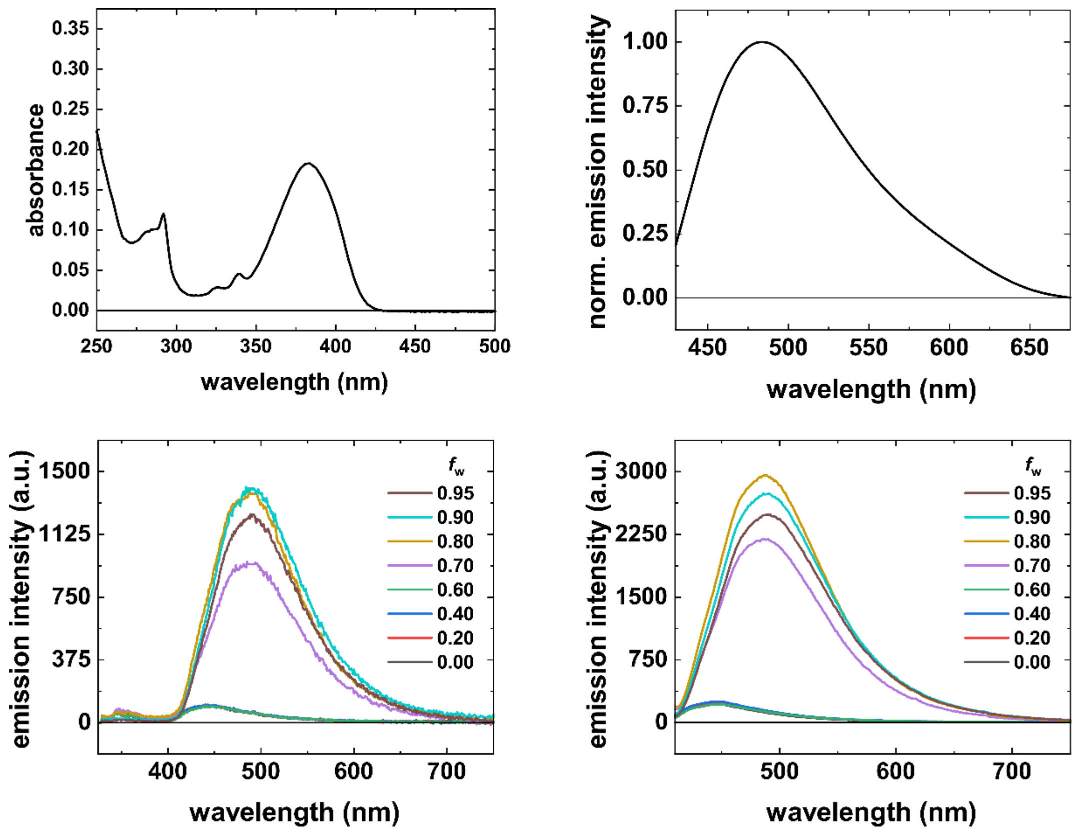

Figure S4: Absorption spectra of **3d** in ethanol (top, left), solid-state emission spectrum (top, right), AIE-related emission spectra of compound **3d** (2nd row) at different excitation wavelengths. (λ_{exc} = 291 nm (bottom left); λ_{exc} = 383 nm (bottom right)).

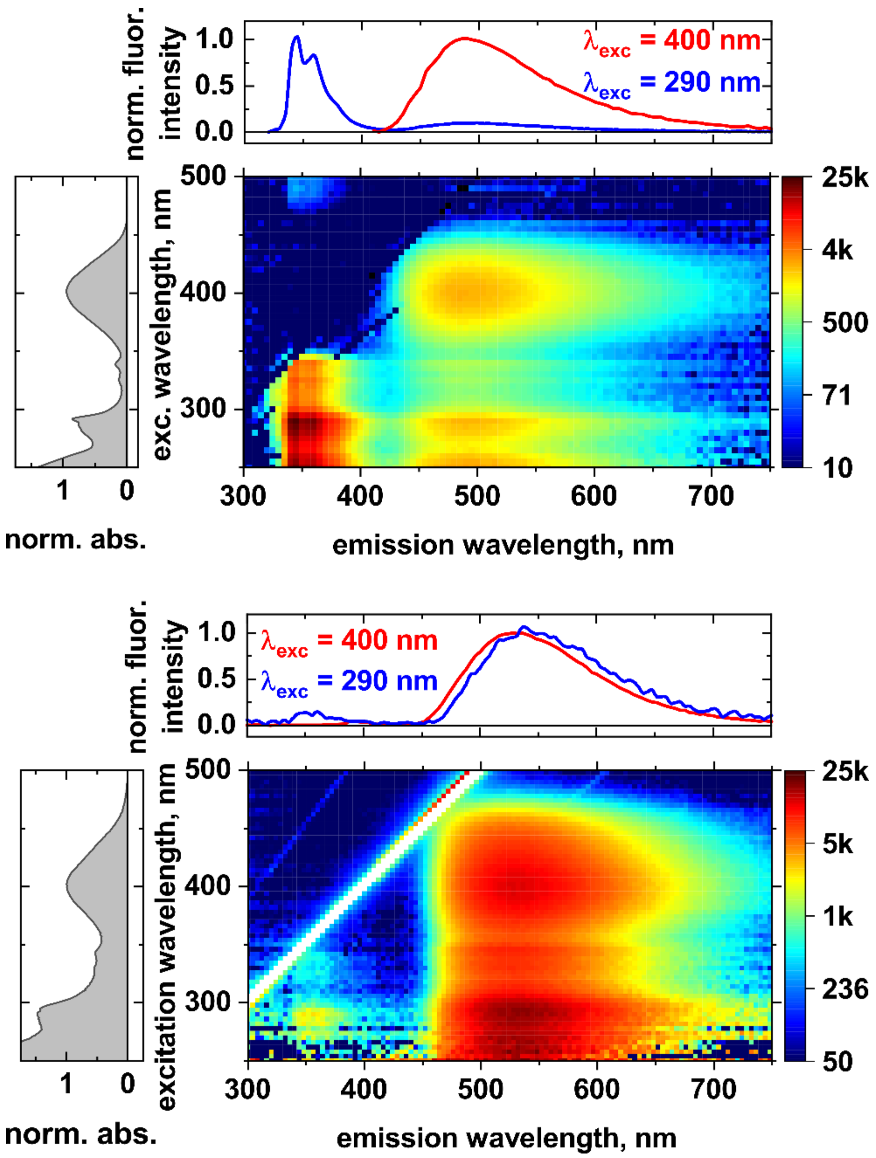

Figure S5. Excitation–emission maps (EEMs) of 3b in ethanol (top, fw = 0) and at high water fraction (bottom, fw_= 0.8), showing the shift from donor-dominated to acceptor-dominated emission upon aggregation (10^{-6} M, T = 298 K).

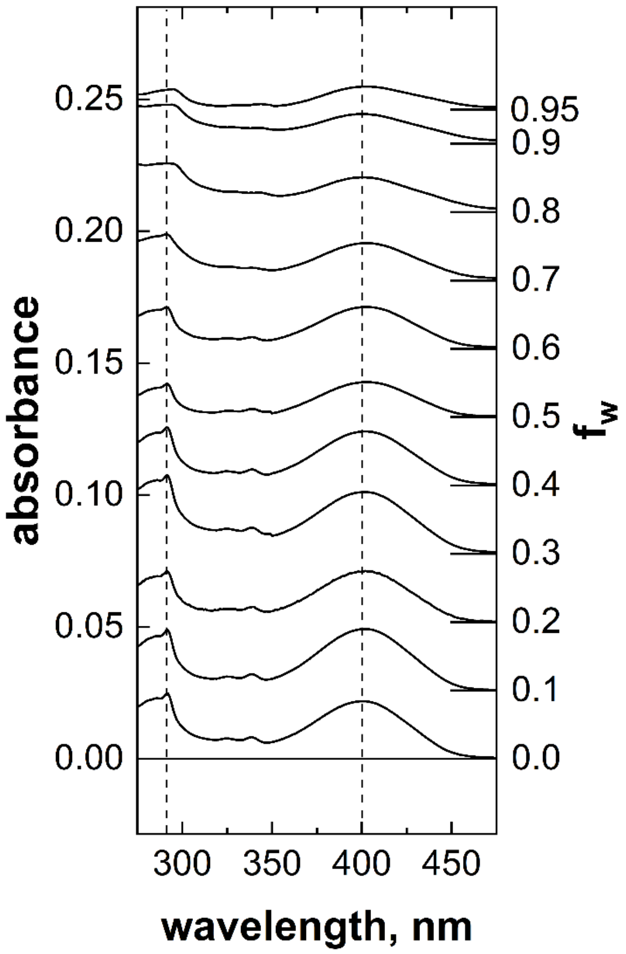

Figure S6. Absorbance spectra of bichromophore **3b** in ethanol/water mixtures with increasing water fraction (fw = 0-0,95). The spectra were recorded at identical dye concentration (10^{-6} M, *T* = 298 K) and are displayed with vertical offsets for better visualization. The dashed lines serve as guideline to the eye to follow the observed changed corresponding to the donor (290 nm) and acceptor moieties (400 nm).

6 Spectral overlap table and calculation note

Table S3. Donor and acceptor model compounds used for the determination of the spectral overlap in bichromophores **3a-3d**.

donor-acceptor molecule	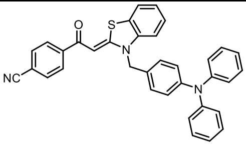 **3a**	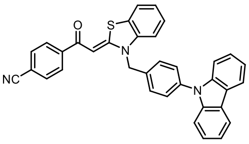 **3b**	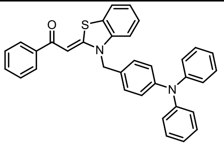 **3c**	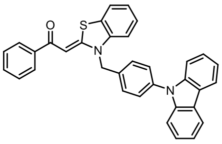 **3d**
donor only model	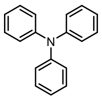	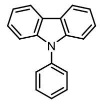	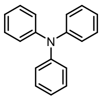	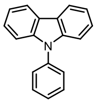
acceptor only model	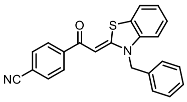 [1]	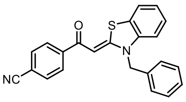 [1]	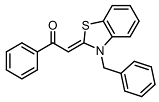 [1]	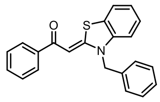 [1]

The spectral overlap analysis was performed using isolated donor and acceptor model compounds representative for **3.** The donor model compounds correspond to phenylcarbazole (PhCarb) or triphenylamine (TPA), which are commercially available and were recorded in ethanol solution, while the acceptor model compounds correspond to the two aroyl-*S*,*N*-ketene acetals, which were reported in ref. 1 and the absorption spectra were recorded in ethanol for this study (see Table S4). The benzyl substituent depicted in the acceptors model compounds does not significantly influence the donor-acceptor spectral overlap.

Table S4. Donor/Acceptor band parameters and spectral overlap integrals:

**Compound**	**Donor λ_{max}^{em} (nm)**	**Donor FWHM (nm)**	**Acceptor λ_{max}^{abs} (nm)**	**Acceptor FWHM (nm)**	**Spectral overlap integral J ($M^{-1}\cdot cm^{-1}\cdot nm^4$)**
**3a**	358	341–382	401	370–430	**$1.93 \cdot 10^{14}$**
**3b**	345	341–368	401	370–430	**$1.03 \cdot 10^{14}$**
**3c**	358	341–382	382	359–404	**$4.99 \cdot 10^{14}$**
**3d**	345	341–368	382	359–404	**$3.32 \cdot 10^{14}$**

The spectral overlap integral J was calculated using the standard wavelength domain expression: $J = \int F_D(\lambda)\, \varepsilon_A(\lambda)\, \lambda^4\, d\lambda$

Where:

- $F_D(\lambda)$ is the donor emission spectrum are-normalized so that $\int F_D(\lambda)\, d\lambda = 1$.
- $\varepsilon_A(\lambda)$ Is the acceptor molar extinction coefficient ($M^{-1}.cm^{-1}$). When the absorbance $A(\lambda)$ was measured, $\varepsilon_A(\lambda) = A(\lambda)/(cl)$ (with c in M and the optical pathlength l in cm).
- The wavelength λ is expressed in nm such that the resulting J has the unit $M^{-1}\cdot cm^{-1}\cdot nm^4$.

7 3b incorporation into polystyrene particles (PSP)

Experimental procedure

Dye-encapsulation in PSP. The encapsulation of compound **3b** into carboxy-functionalized polystyrene particles (PSP, 200 nm) was performed using a swelling procedure.[10] The dyes were initially dissolved in THF (10^{-4} M), and 100 µL of this solution was added to 600 µL of an aqueous PSP suspension (0.5 w%). The mixture underwent a 1-hour incubation cycle consisting of alternating 15-minute shaking and 2-minute sonication periods. Following incubation, 900 µL of deionized water was added, and the suspension was centrifuged at 21,130 rcf for 25 min. The particles were then washed twice with deionized water and resuspended in water using ultrasonication.

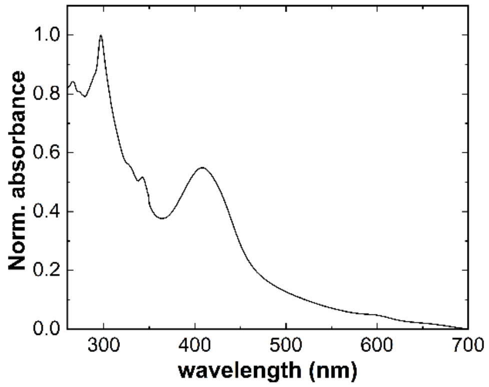

Figure S7. Normalized extinction spectrum of bichromophore **3b** encapsulated in PSP. The spectrum was obtained after background correction for particle scattering using unloaded PSP as a reference and hence is dominated by the absorption characteristics of **3b** in the apolar polymer matrix.

8 Crystal structure data

Table S5: Crystal structure data for **3c**.

Structure	3c
Empirical formula	$C_{34}H_{26}N_2OS$
Moiety formula	$C_{34}H_{26}N_2OS$
Formula weight [g/mol]	510.63
Temperature [K]	150(1)
Crystal system	monoclinic
Space group	$P2_1/n$
a [Å]	16.25650(10)
b [Å]	8.23820(10)
c [Å]	19.28180(10)
α [°]	90
β [°]	90.9410(10)
γ [°]	90
Volume [Å^3]	2581.95(4)
Z	4
$\rho_{calb\ c}$ [g/cm^3]	1.314
μ [mm^{-1}]	1.347
F(000)	1072.0
Crystal size [mm]	0.18×0.09×0.07
Radiation	Cu Kα (λ = 1.54184)
2Θ range for data collection [°]	5.436 to 158.544
Index ranges	-18 ≤ h ≤ 20, -10 ≤ k ≤ 10, -18 ≤ l ≤ 24
Reflections collected	38605
Independent reflections	5340 [R_{int} = 0.0326, R_{sigma} = 0.0191]
Data/restraints/parameters	5340/0/367
Goodness of fit on F^2	1.071
Final R indexes (I ≥ 2σ (I))	R_1 = 0.0311, wR_2 = 0.0812
Final R indexes (all data)	R_1 = 0.0336, wR_2 = 0.0831
Largest diff. peak/hole [e Å^{-3}]	0.18/-0.33

The merocyanine is only slightly twisted with an angle of 19.88° between the aroyl ring and the benzo part of the benzothiazole terminus. The crystal packing (Figure S8) reveals a back-to-back alignment of the planarized merocyanine with a centroid to centroid distance between the two rings $d_{centroid}\ldots d_{centroid}$ = 3.925 Å, suggesting π-π-stacking (Figure S9). The distances and angles were determined using the program Mercury. The twisting angle of the merocyanine was measured between two planes, one plane was calculated from the six carbon atoms of the aroyl component, one was calculated from the six carbons of the benzo part of the

benzothiazole. To measure the distance between two merocyanine units, planes were calculated from the C, O, N, and S atoms of the merocyanines, and the distance between the two planes was measured (Figure S9).

The triarylamino blue emitters are positioned almost orthogonally, interlocking with the triphenylamine units of neighboring bichromophores. Due to the molecular structure, no strong intermolecular interactions are expected to occur, as visualized by the Hirshfeld surface (Figure S10).[6] As evident from the fingerprint plot (Figures S11-14),[8] the π - π -stacking (C-C contacts, 4.1%) and C-H contacts (30.1%) contribute to the crystal packing, along with close packing effects. These are represented by numerous H-H contacts (53.2%), which are expressed as a peak in the fingerprint plot (bottom left). However, the carbonyl group does not lie within the sum of the van der Waals radii.[9] The absence of strong intermolecular interactions is consistent with the low melting point (102 °C) of dye **3c**.

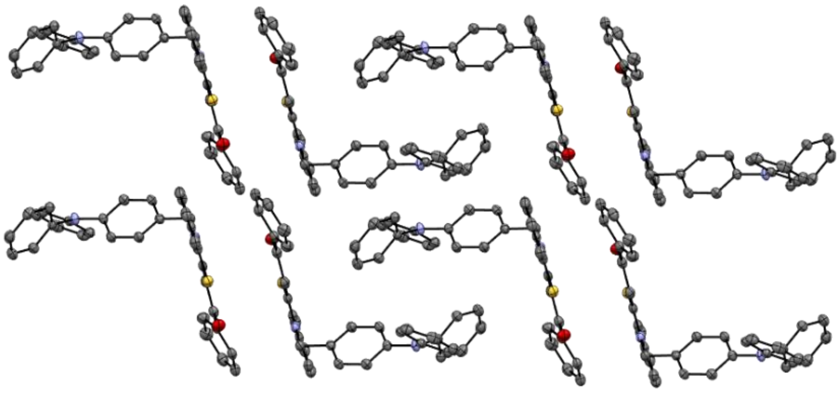

Figure S8: Crystal packing (hydrogen atoms omitted for clarity.

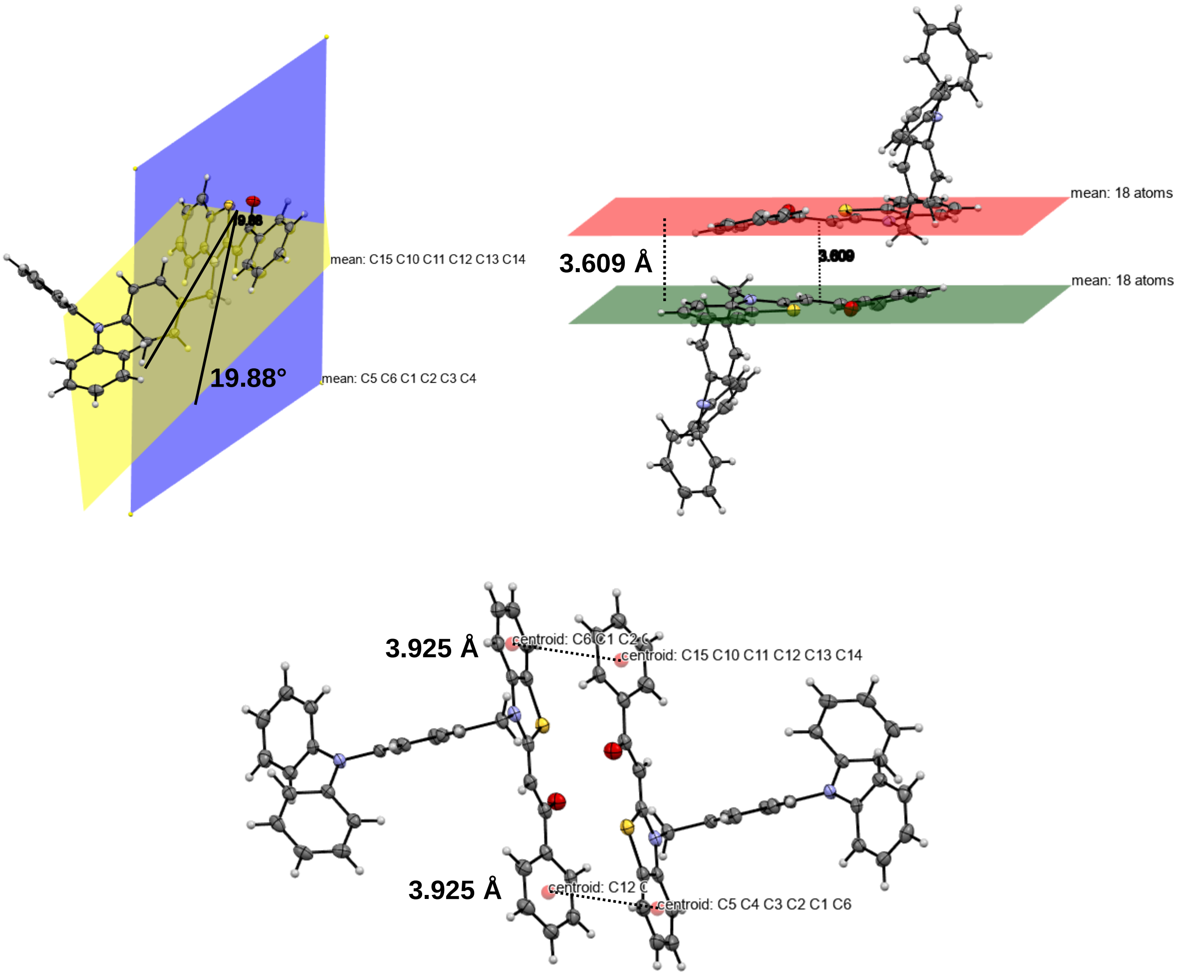

Figure S9: Angle (top, left) and distance (top, right and bottom) measurements of compound **3c**.

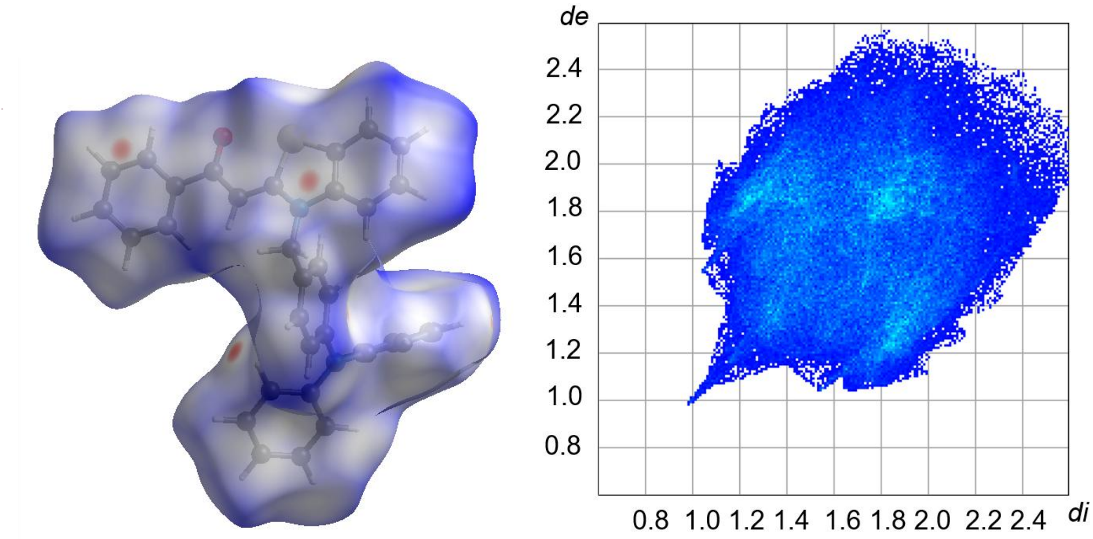

Figure S10: Hirshfeld surface of **3c** mapped with d_{norm} (left) and fingerprint plot for **3c**.

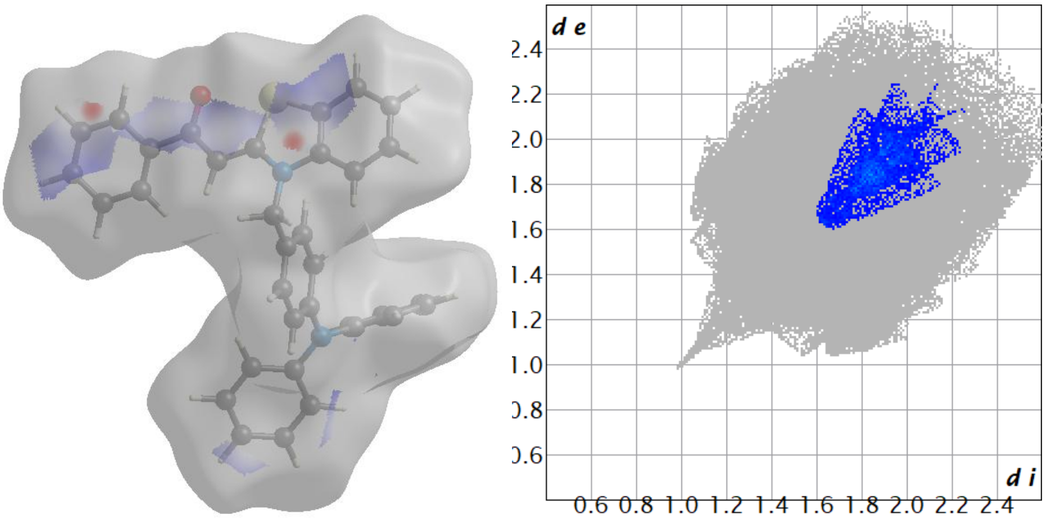

Figure S11: Fingerprint plot contributions from C-C contacts (4.1%) for compound **3c**.

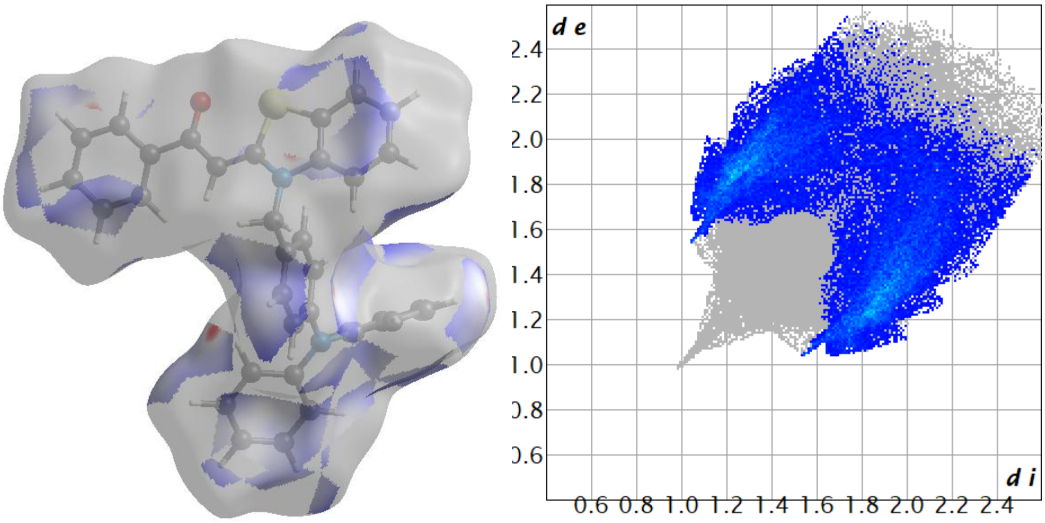

Figure S12: Fingerprint plot contributions from C-H contacts (30.1%) for compound **3c**.

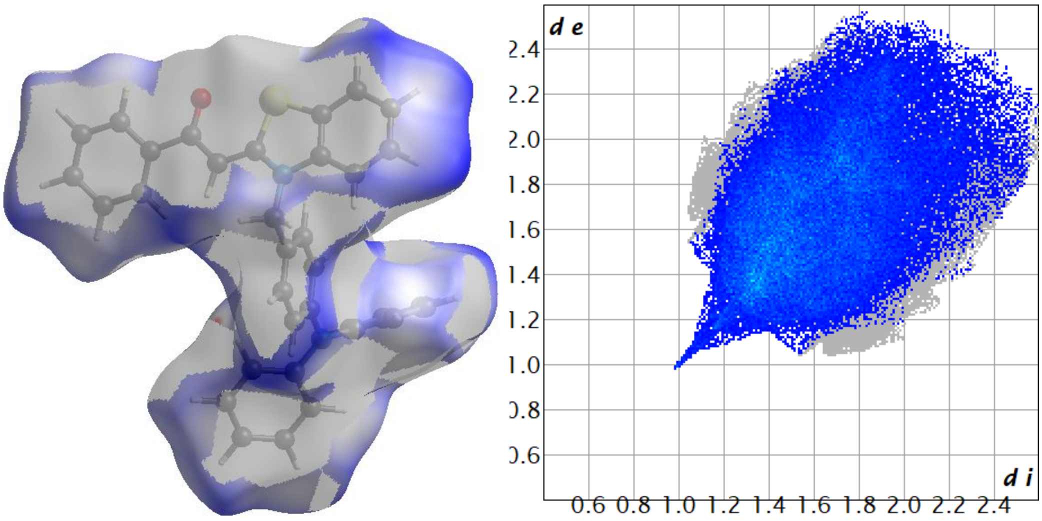

Figure S13: Fingerprint plot contributions from H-H contacts (53.2%) for compound **3c**.

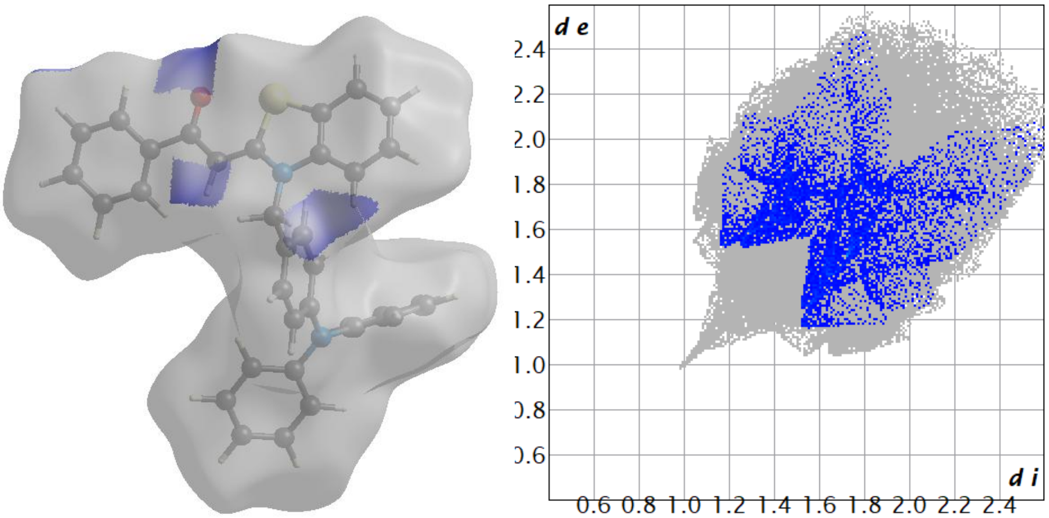

Figure S14: Fingerprint plot contributions from O-H contacts (5.5%) for compound **3c**.

9 Literature

1. M. Hesse, H. Meier and B. Zeeh, *Spektroskopische Methoden in der organischen Chemie*, Georg Thieme Verlag, 2005.
2. *CrysAlisPRO*, Oxford Diffraction / Agilent Technologies UK Ltd., Yarnton, Oxfordshire, England, 2014.
3. O. V. Dolomanov, L. J. Bourhis, R. J. Gildea, J. A. Howard and H. Puschmann, *J. Appl. Crystallogr.*, 2009, **42**, 339-341.
4. G. M. Sheldrick, *Acta. Cryst.*, 2015, **71**, 3-8.
5. G. M. Sheldrick, *Acta. Cryst.*, 2008, **64**, 112-122.
6. P. R. Spackman, M. J. Turner, J. J. McKinnon, S. K. Wolff, D. J. Grimwood, D. Jayatilaka and M. A. Spackman, *J. Appl. Crystallogr.*, 2021, **54**, 1006-1011.
7. J. Krenzer and T. J. Müller, *Beilstein J. Org. Chem.*, 2025, **21**, 1201-1206.
8. M. A. Spackman and J. J. McKinnon, *CrystEngComm*, 2002, **4**, 378-392.
9. A. v. Bondi, *J. Phys. Chem.*, 1964, **68**, 441-451.
